# Physiological and genomic evidence that selection on the transcription factor *Epas1* has altered cardiovascular function in high-altitude deer mice

**DOI:** 10.1371/journal.pgen.1008420

**Published:** 2019-11-07

**Authors:** Rena M. Schweizer, Jonathan P. Velotta, Catherine M. Ivy, Matthew R. Jones, Sarah M. Muir, Gideon S. Bradburd, Jay F. Storz, Graham R. Scott, Zachary A. Cheviron

**Affiliations:** 1 Division of Biological Sciences, University of Montana, Missoula, Montana, United States of America; 2 Department of Biology, McMaster University, Hamilton, ON, Canada; 3 Ecology, Evolutionary Biology, and Behavior Graduate Group, Department of Integrative Biology, Michigan State University, East Lansing, Michigan, United States of America; 4 School of Biological Sciences, University of Nebraska, Lincoln, Nebraska, United States of America; University of Chicago, UNITED STATES

## Abstract

Evolutionary adaptation to extreme environments often requires coordinated changes in multiple intersecting physiological pathways, but how such multi-trait adaptation occurs remains unresolved. Transcription factors, which regulate the expression of many genes and can simultaneously alter multiple phenotypes, may be common targets of selection if the benefits of induced changes outweigh the costs of negative pleiotropic effects. We combined complimentary population genetic analyses and physiological experiments in North American deer mice (*Peromyscus maniculatus*) to examine links between genetic variation in transcription factors that coordinate physiological responses to hypoxia (hypoxia-inducible factors, HIFs) and multiple physiological traits that potentially contribute to high-altitude adaptation. First, we sequenced the exomes of 100 mice sampled from different elevations and discovered that several SNPs in the gene *Epas1*, which encodes the oxygen sensitive subunit of HIF-2α, exhibited extreme allele frequency differences between highland and lowland populations. Broader geographic sampling confirmed that *Epas1* genotype varied predictably with altitude throughout the western US. We then discovered that *Epas1* genotype influences heart rate in hypoxia, and the transcriptomic responses to hypoxia (including HIF targets and genes involved in catecholamine signaling) in the heart and adrenal gland. Finally, we used a demographically-informed selection scan to show that *Epas1* variants have experienced a history of spatially varying selection, suggesting that differences in cardiovascular function and gene regulation contribute to high-altitude adaptation. Our results suggest a mechanism by which *Epas1* may aid long-term survival of high-altitude deer mice and provide general insights into the role that highly pleiotropic transcription factors may play in the process of environmental adaptation.

## Author summary

Adaptation often requires coordinated evolutionary changes across multiple dynamic systems to maintain physiological function. For example, high-altitude habitats place a premium on tissue-oxygen delivery to cope with limited oxygen availability (hypoxia). Circulatory O_2_ transport is regulated dynamically, changing on the order of seconds, and results from several interacting physiological processes. The mechanisms of adaptation in such complex phenotypes are poorly understood. One promising candidate is the gene *Epas1*, which encodes a transcription factor that regulates physiological responses to hypoxia. We used population genomic analyses and physiological assays to explore the connections between *Epas1* genetic variation and physiological function in high-altitude deer mice, which exhibit evolutionary adaptations to hypoxia. We identified a mutation in *Epas1* that is associated with variation in cardiovascular function: the predominant variant at high altitude is associated with the maintenance of an elevated heart rate under hypoxia and with differences in the expression of genes that influence heart rate and are regulated by *Epas1*. Our population genomic analyses demonstrated that *Epas1* exhibits a signature of natural selection at high altitude, suggesting that these phenotypic effects influence Darwinian fitness. Our results suggest that adaptation in complex and dynamic traits may be attributable to relatively simple genetic changes.

## Introduction

Adaptive evolution often involves changes in multiple phenotypes across interacting biological pathways. How such multi-trait adaptations are produced by natural selection is an open question that requires connecting genetic variation to organismal function and fitness [1]. One promising mechanism involves functional modification of transcription factors. Because transcription factors coordinate the expression of suites of genes, they may allow for the simultaneous alteration of multiple phenotypes, making them common targets of selection [2–4]. However, mutational changes in transcription factors often have negative pleiotropic effects, which may limit the role of such changes in environmental adaptation [5,6]. If pleiotropic constraints are common, then mutations in downstream target genes may be expected to play a more prominent role in local adaptation [5,6].

Animals adapted to high-altitude (>3,000 m a.s.l.) [7] represent a unique system to understand the role of transcription factors in multi-trait adaptation. Coping with extreme hypoxia (low O_2_ availability) and cold requires coordinated changes in interacting physiological pathways [8–10], including steps of the O_2_ transport cascade that ensure O_2_ supply matches demand. Many of these responses to hypoxia are coordinated by a single family of transcription factors, the hypoxia inducible factors (HIF 1–3) [11]. In particular, the gene *Epas1*, which encodes the O_2_-sensitive α subunit of HIF-2, has been the repeated target of selection in indigenous high-altitude human and non-human populations [8,12–15]. In many ways, this pattern of repeated selection is surprising: although acute activation of HIFs lead to beneficial changes in O_2_ homeostasis (e.g, *via* ventilatory acclimatization [16] and angiogenesis [17]), chronic HIF activity is often linked to high-altitude disease [10]. Thus, modification of HIF signaling may be constrained by antagonistic pleiotropy.

Determining the extent of pleiotropic constraint requires an understanding of the phenotypic effects of naturally segregating HIF variants. Studies in indigenous Tibetan humans, for example, have linked allelic variation at *Epas1* to the maintenance of normal blood hemoglobin content [8,13] and blood concentrations of erythropoietin (which stimulates red blood cell production) under conditions of environmental hypoxia [18]. This maintenance of hemoglobin content under hypoxia is also associated with a missense variant in *Egln1* that covaries with *Epas1* genotype in Tibetans [18] and promotes HIF degradation under hypoxia [19]. However, follow-up studies found that *Epas1* genotype did not have a statistically significant influence on breathing or pulmonary function within Tibetans living at low elevation, although there were pronounced differences in several cardiorespiratory phenotypes between Tibetans and Han Chinese [18]. Nevertheless, a range of other respiratory and cardiovascular system responses to chronic hypoxia are influenced by HIF-2 signaling, including the hypoxic ventilatory response [16,20], catecholamine synthesis by the adrenal gland [21], and others [22], and it remains unclear if these phenotypes have been altered by selection on *Epas1*, particularly in other highland taxa. A more detailed understanding of the phenotypic effects of HIF variation is needed in order to ascertain the general role of regulatory pleiotropy in multi-trait physiological adaptation to high altitude.

We used the North American deer mouse (*Peromyscus maniculatus*) to examine links between genetic variation in HIFs and multiple physiological adaptations to high altitude. Within the continental U.S., deer mice are distributed across an altitudinal range of ~4500 m, and have consequently emerged as a prominent model for studies of the mechanisms of adaptation [23–31]. Deer mice native to the Rocky Mountain highlands have evolved a unique physiology that includes suites of adaptations linked to known phenotypes related to HIF signaling (e.g. hematological function, heart rate, tissue capillarity, and metabolic fuel use) [25,26,31–39]. Given the evidence for multi-trait physiological adaptation to high altitude in deer mice, and the recent indications that *Epas1* has been a repeated target of natural selection in multiple highland specialists, we hypothesized that adaptive phenotypic variation is attributable, at least in part, to naturally segregating genetic variation in genes that encode HIFs.

## Results

### *Epas1* genotype varies with altitude in deer mice

In order to examine altitudinal patterns of allele frequency variation of the genes encoding HIFs, and to put these patterns into a broader genomic context, we sequenced the exomes of 37 lowland mice from Lincoln, NE (430 m a.s.l.), and 48 highland mice from Mt. Evans, CO (4350 m a.s.l). Fifteen mice from a lowland population in Merced County, CA (~320 m a.s.l.), were included to infer polarity of DNA changes in highland mice. All exomes were sequenced using a custom Nimblegen probe set targeting exons from 25,246 nuclear genes (see Materials and Methods). Captured exomes were paired-end sequenced on an Illumina HiSeq 4000 and mapped to a reference genome (NCBI GCA_000500345.1 Pman_1.0). The final set of quality-filtered sites consisted of 5,182,530 high-quality bi-allelic variants sequenced at approximately 18X coverage (S1 Fig). Analyses of population genetic structure (using PCA [40] and Admixture [41]), revealed that all three populations were genetically distinguishable (S2 Fig and S3 Fig). Pairwise F_ST_ values (estimated with Weir’s Theta [42]) between Mt. Evans and Lincoln were 0.025± 3.16e-5 (mean±SEM), between Mt. Evans and Merced were 0.025±6.54e-5, and between Lincoln and Merced were 0.044±8.00e-5.

**Fig 1:**
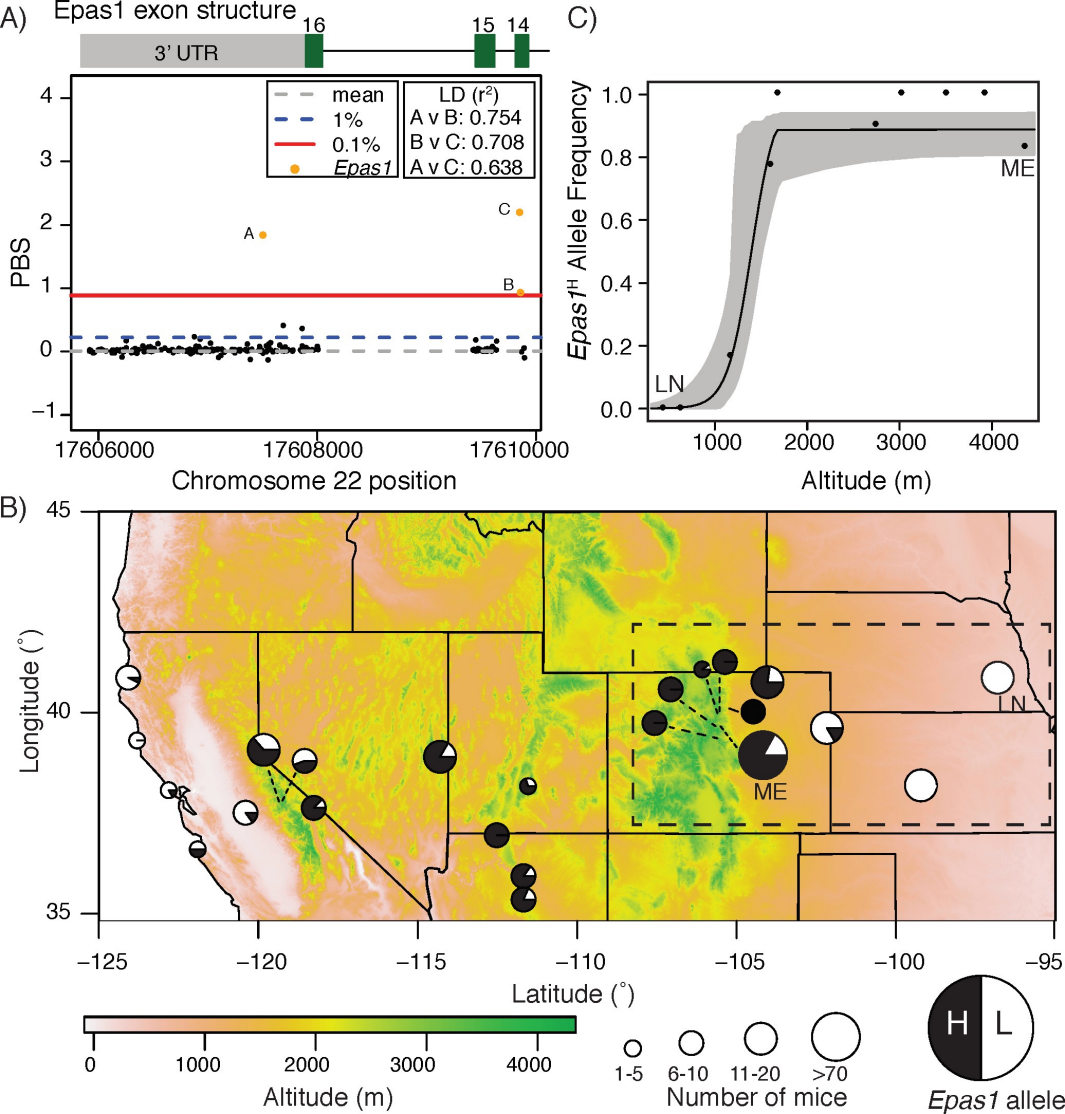
*Epas1* is an exome-wide outlier under spatially varying selection in *P*. *maniculatus* along altitudinal gradients. A) Manhattan plot of PBS values for all SNPs (black dots) located within the last three exons of *Epas1* (exon numbers provided above schematic). Exome-wide values for mean, top 1%, and top 0.1% percentile PBS values are shown, and three outlier SNPs in *Epas1* are highlighted in orange (see key). Pairwise linkage disequilibrium estimates (measured with the squared correlation coefficient, r^2^) for each SNP pair are provided. B) Geographic variation in ^Thr^755^Met^ *Epas1* allele frequency for 23 populations in the Rocky Mountains and Great Plains, USA. Pie charts are shaded according to frequency of high-altitude or low-altitude allele, with size indicating number of mice sampled (see key). C) Clinal variation in ^Thr^755^Met^ *Epas1* allele frequency for 10 *P*. *maniculatus* populations sampled along a 4500 m altitudinal cline from the Great Plains of Nebraska to the Rocky Mountains in Colorado. In (B) and (C), Mt. Evans (ME) and Lincoln (LN) populations are labeled. Dashed box in (B) shows populations chosen for assessing clinal variation in (C). See S1 Table for details on sampling location and *Epas1* allele frequencies.

Based on these results, we calculated the population branch statistic (PBS [13]) for each single nucleotide polymorphism (SNP) to identify variants that exhibit extreme allele frequency changes in the highland population (Mt. Evans) relative to both lowland populations (Lincoln and Merced) (S4 Fig). Among the upper 0.1% of the PBS distribution, the only SNPs located in HIF genes were three SNPs located in the HIF-2α gene *Epas1* (Fig 1A, S5 Fig); one of these SNPs was located in the 3’UTR, one was a non-synonymous polymorphism located at site 755 in the 14^th^ exon that changed threonine to methionine (^Thr^755^Met^), and one was a synonymous polymorphism also located in the 14^th^ exon. The highest-ranking SNP of these three was the non-synonymous, polarity-altering ^Thr^755^Met^ polymorphism (PBS upper 0.1%; Fig 1A). Due to significant linkage disequilibrium between alleles at the three closely linked *Epas1* SNPs (Fig 1A), and because there were no SNPs in any of the genes that encode HIF-1α or HIF-3α in the upper 0.1% of the empirical PBS distribution, we focused our subsequent analyses on the ^Thr^755^Met^ mutation in *Epas1*.

To more broadly assess the relationship between *Epas1* ^Thr^755^Met^ and elevation, we genotyped an additional 266 deer mice collected from 23 sites across the western U.S. (S1 Table). We found that the Met-755 allele (henceforth called the *Epas1*^H^ allele) is significantly and positively correlated with altitude (r^2^ = 0.589, p<0.001; Fig 1B; S6 Fig). For a single altitudinal transect connecting Lincoln to Mt. Evans, variation in *Epas1* allele frequency is best explained as a sigmoidal cline centered at 1399.5 m a.s.l. (95% CI 1192.99–1493.01 m a.s.l.) (Fig 1C). Notably, the *Epas1* cline is similar in shape, width, and center to that of ß-globin [43] (S7 Fig), a locus known to be under selection in high-altitude deer mice [28,43,44]. To infer character polarity of the amino acid change, we genotyped mice from nine additional *Peromyscus* species, including *P*. *keeni*, *P*. *melanotis*, *P*. *hylocytes*, *P*. *attwateri*, *P*. *melanophrys*, *P*. *eremicus*, *P*. *polionotus*, *P*. *leucopus*, as well as an outgroup rodent species, *Reithrodontomys montanus*. This broader phylogenetic sampling suggests that the high-altitude variant, *Epas1*^H^, is the derived allele within the *P*. *maniculatus* subclade (S2 Table; SI Results).

### *Epas1* genotype is associated with physiological traits that influence oxygen homeostasis

We tested for physiological effects of allelic variation at *Epas1* using deer mice captured on the summit of Mt. Evans. *Epas1*^H^ and *Epas1*^L^ alleles segregate in this population, occurring at frequencies of 0.83 and 0.17, respectively (Fig 1), which allowed us to isolate the effects of genotype on an otherwise randomized genomic background. We developed a restriction digest protocol to genotype mice in the field (see Methods), and then subjected mice of known genotype to a series of tests to characterize variation in traits that influence O_2_ homeostasis under hypoxia. We used whole-body plethysmography and pulse oximetry during a step-wise hypoxia exposure [20 min at 21 (sea-level), 12 (equivalent to Mt. Evans altitude), 10, 8, and 6 kPa] to test for genotypic effects on acute respiratory and cardiovascular responses to hypoxia: breathing frequency and tidal volume, rate of O_2_ consumption (VO_2_), arterial O_2_ saturation, and heart rate. Forty-eight hours following plethysmography and pulse oximetry, mice were exposed to deep hypoxia (6 kPa) for 2 hours and then euthanized. This duration of exposure to 6 kPa O_2_ was chosen in order to strongly stimulate the HIF-induced transcriptional response, as supported by previous observations that this hypoxic treatment strongly induces the expression of *Vegfa*, a key HIF target, in many organisms [44]. Blood, heart, adrenal glands, lungs, and gastrocnemius muscle were sampled for transcriptomic profiling and/or for morphological and histological analysis.

We did not detect significant effects of allelic variation at *Epas1* on some traits that are indicative of chronic exposure to hypoxia or that otherwise influence O_2_ transport and utilization. Unlike in studies of indigenous Tibetans [12], *Epas1* genotype did not affect hematocrit or hemoglobin concentration at high altitude (S3 Table, S4 Table). Similarly, we did not detect any genotypic differences in the fiber size (S8 Fig, S9 Fig) or the activities of oxidative enzymes in the gastrocnemius muscle (S5 Table, S10 Fig). There were trends toward reduced muscle capillarity in individuals that were homozygous for the lowland allele (*Epas1*^L/L^), and reduced lactate dehydrogenase activity in the muscle of individuals that were homozygous for the highland allele (*Epas1*^H/H^), but these differences were not statistically significant when compared to all other genotypes (S8 Fig, S9 Fig, S10 Fig).

**Fig 2:**
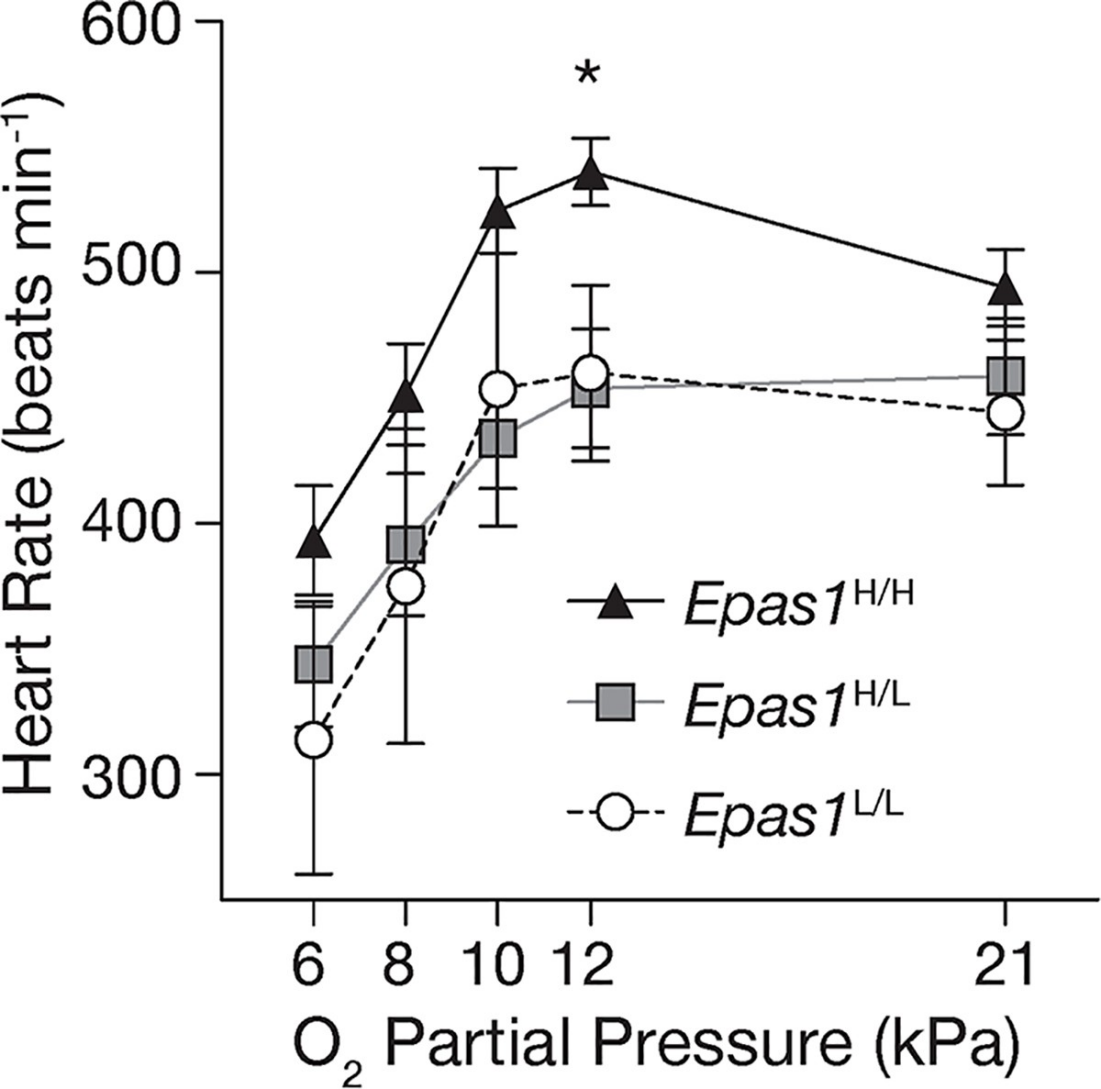
Deer mice that were homozygous for the highland *Epas1* variant exhibited higher heart rates when exposed to environmentally realistic levels of hypoxia at 4300 m altitude (12 kPa O_2_). Measurements were made using a MouseOx Plus collar. * Significant main effect of *Epas1* genotype in a mixed linear model. n = 26 *Epas1*^H/H^, n = 13 *Epas1*^H/L^, and n = 4 *Epas1*^L/L^ variants.

With respect to respiratory and cardiovascular function under acute hypoxia, we also did not detect any genotypic differences in breathing (total ventilation, breathing frequency, tidal volume), VO_2_, body temperature depression, or pulmonary O_2_ extraction (S11 Fig, S12 Fig, S6 Table). However, *Epas1* genotype did affect heart rate under ecologically relevant levels of hypoxia: resting heart rates in normoxia (21 kPa O_2_) were similar across genotypes, but individuals that were homozygous for the highland allele (*Epas1*^H/H^) maintained higher resting heart rates during hypoxia exposure compared to the other two genotypes (*Epas1*^L/-^) (Fig 2). We detected a significant main effect of genotype on heart rate (F_2,148_ = 3.8; *p* = 0.03), but a non-significant interaction (p>0.05), suggesting that *Epas1*^H/H^ mice generally maintained higher resting heart rates. The genotypic difference in heart rate was most pronounced at 12 kPa O_2_, which approximates PO_2_ at the summit of Mt. Evans (Fig 2). However, the magnitude of the increase in heart rate from normoxia to 12 kPa O_2_ was greatest in *Epas1*^H/H^ mice (S13 Fig), suggesting that *Epas1* genotype may have influenced the heart rate response to hypoxia at the environmental PO_2_ that are realistic at the high-altitude field site. Heart rate did not differ between heterozygotes and homozygotes for the lowland allele at any PO_2_ (Fig 2). We observed a steady decline in resting heart rate for all three genotypes as the level of hypoxia increased at PO_2_ below 10 kPa (main effect of PO_2_: F_4,148_ = 19.741, P<0.001), likely as a consequence of the depression in VO_2_ and body temperature under extreme hypoxia (S12 Fig).

### *Epas1* genotype affects the regulation of HIF target and catecholamine genes

We used an RNA-seq approach (TagSeq [45]) to test whether *Epas1* genotypes differ in gene regulation in two tissues affecting heart rate under hypoxia: the adrenal gland (which affects heart rate and vasoconstrictive responses by secreting catecholamines) and the left ventricle of the heart. For each tissue we measured transcriptome-wide patterns of gene expression, and the expression of two candidate gene sets: HIF target genes and genes involved in catecholamine biosynthesis, secretion, and signaling (S7 Table). Full results of candidate gene differential expression analysis are available in the online supplement (S8 Table). Overall, this analysis revealed a significant association between *Epas1* genotype and the regulation of genes in HIF- and catecholamine-related pathways in both tissues.

**Fig 3:**
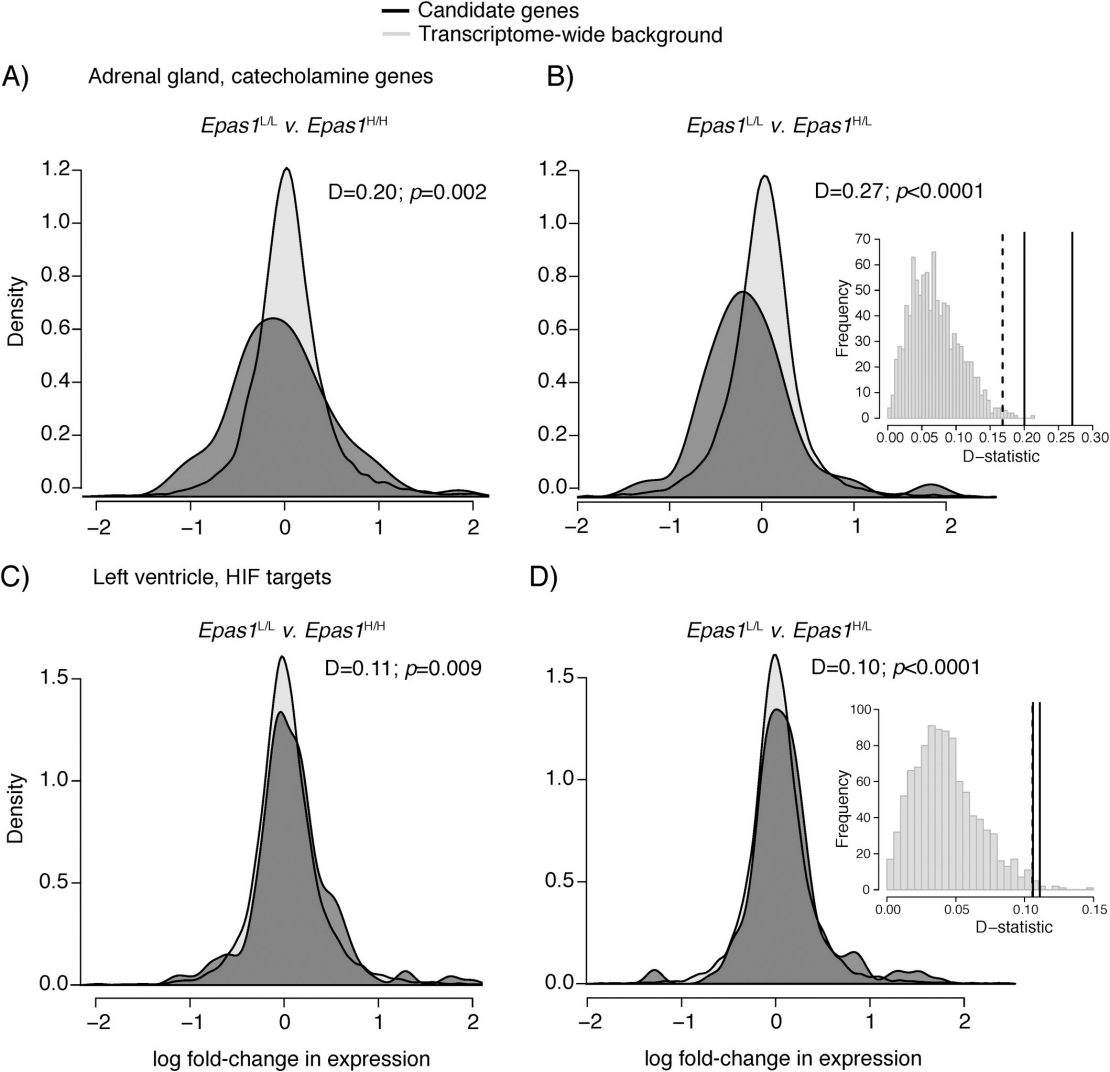
*Epas1* genotype affects the regulation of HIF and catecholamine genes. Distribution of log fold-change values between *Epas1*^L/L^ v. *Epas1*^H/H^ and *Epas1*^H/L^ for candidate genes (black) compared the the background transcriptome-wide distribution (grey) for adrenal catecholamine genes (A-B) and left ventricle HIF targets (C-D). Insets show the distribution of K-S test D-statistics for 1,000 randomly permuted datasets tested against the transcriptome-wide background. Dashed indicated the 99% quantile of the null distribution, while solid lines indicate the observed D-statistic for each comparison.

In the adrenal gland, we detected a subtle but significant shift toward reduced expression of candidate genes in mice possessing the high-altitude *Epas1* allele (Fig 3A and 3B). Kolmogorov-Smirnov (K-S) tests revealed significant differences in the distribution of log fold-change values for each comparison of *Epas1*^L/L^ vs. *Epas1*^H/L^ and *Epas1*^H/H^ in catecholamine-associated genes (Fig 3A and 3B), but not HIF targets (*p*>0.05). Specifically, log-fold change values were significantly more negative for candidate genes in *Epas1*^H/-^ mice compared to the transcriptome-wide background (Fig 3A and 3B), indicating a reduced expression of these genes in mice that carry *Epas1*^H^ alleles. To test the robustness of this result, we generated a null distribution of K-S test D-statistics by drawing 1000 random gene sets that were equal in size to the candidate catecholamine gene set (*n* = 79). This randomization procedure demonstrates that the D-statistics calculated for both genotypic comparisons were above the 99% quantile of the null distribution (Fig 3B inset). The pattern of down-regulation of catecholamine-related genes is consistent with recent findings that, compared to low-altitude deer mice from Nebraska, high-altitude mice express lower levels of DOPA decarboxylase in the adrenal medulla and exhibit reductions in catecholamine release from adrenal chromaffin cells [46]. Moreover, HIF-2α has been shown to be a positive regulator of catecholamine synthesis in adrenal chromaffin cells of rats [21], suggesting that the high-altitude *Epas1* variant leads to a direct reduction in gene expression of enzymes involved in catecholamine biosynthesis, and thereby reduces circulating catecholamine levels.

In a related analysis, we found that positive regulators of catecholamine processes (*n* = 15) were downregulated in mice possessing the high-altitude allele (mean log fold-change between *Epas1*^H/H^ and *Epas1*^L/L^: -0.04 ± 0.17), relative to genes known to be negative regulators of catecholamines (*n* = 13; mean log fold-change = 0.23 ± 0.19). This trend, though not statistically significant (*p*>0.05) is consistent with the hypothesis that possessing the high-altitude *Epas1* allele is associated with a downregulation of genes that positively influence catecholamine processes. Due to the small size of the subset of genes for which a directional influence could be established, and the lack of statistical significance, these results should be viewed with caution.

In contrast to the adrenal gland, candidate gene expression in the left ventricle was significantly higher in mice possessing *Epas1*^*H*^ alleles, but this effect was subtle and only significant for HIF target genes (Fig 3C and 3D). K-S tests revealed a significant shift toward more positive distribution of log fold-change values in *Epas1*^H/H^ (Fig 3C) and *Epas1*^H/L^ (Fig 3D) mice. Randomization procedures indicated that the empirical D-statistic falls outside of the 99% quantile of the null distribution for both comparisons (Fig 3D). We note that several genes in the top 10% of the distribution of log fold-change in expression (adrenomedullin [*Adm*], endothelin [*Edn1*], and atrial natriuretic peptide [*Nppa*]; S8 Table) are known effectors of vasodilation [47–49], suggesting that improved O_2_ supply to cardiac tissue may be positively influenced by *Epas1* genotype.

Importantly, none of the genes in these pathways were differentially expressed among genotypes after correcting for multiple testing in either tissue (FDR >0.05), nor were any other genes in either transcriptome. This result suggests that effects of *Epas1* genotype on the regulation of any single gene are weak in these tissues. We note that the O_2_ pressure (6 kPa) used to stimulate gene expression was strong, and led to uniform metabolic depression across genotypes (Fig 2) potentially masking genotypic differences in expression that may exist at less extreme levels of hypoxia. Future research will examine whether 12 kPa O_2_, the level leading to the largest genotypic differences in heart rate (Fig 2), elicits greater genotypic difference in the expression of HIF and catecholamine target genes, or potentially other unknown pathways. Nevertheless, our results demonstrate that the combined effects of multiple subtle, but concerted, shifts in expression of HIF target genes and genes in catecholamine-related pathways were detectable among genotypes.

### Hypoxia-related genes, including *Epas1*, are targets of positive selection in highland deer mice

To formally test for a history of spatially varying selection on *Epas1* polymorphisms, we performed a demographically-corrected selection scan. We first estimated effective population sizes (*N*_*e*_), divergence times (*T*), and pairwise migration rates (*m*) for two pairs of populations (Mt. Evans-Lincoln and Mt. Evans-Merced) by modeling the folded two-dimensional site frequency spectra (2D-SFS) derived from variation at synonymous SNPs using *∂a∂i* [50]. The best-fitting demographic model (S14 Fig) allowed for variation in gene flow among loci by including two symmetrical migration rate parameters applied to proportions *P* and *1-P* of loci, following [51], which produced significantly better fit to the data (*p* = 0; adjusted likelihood ratio test using the Godambe Information Matrix [52]). For Mt. Evans and Merced, we estimated that approximately 84.2% of SNPs experience a relatively high migration rate (2.07 migrants/generation), while the remaining SNPs experience a substantially lower migration rate (0.08 migrants/generations). Similarly, between Mt. Evans and Lincoln, we estimate that approximately 86.4% of SNPs experience a migration rate of ~1.75 migrants/generation while remaining SNPs have a rate of ~0.08 migrants/generation. Under these models, we estimated effective population sizes of approximately 368,000 (95% CI: 266,977–470,061) for Mt. Evans, 220,000 (95% CI: 170,878–270,767) for Lincoln, and 371,000 (95% CI: 266,871–477,310) for Merced populations (S9 Table). The inferred split time between Mt. Evans and Lincoln populations was approximately 217,000 generations ago (95% CI: 180,998–254,122 generations ago) and the inferred split between Mt. Evans and Merced was 190,000 generations ago (95% CI: 127,569–253,390 generations ago) (S9 Table).

We established a null PBS distribution by simulating 500,000 neutral SNPs (85% with the high migration rates and 15% with the low migration rates) across Mt. Evans, Merced, and Lincoln populations in *msms* [53] under our estimated demographic model, assuming no direct gene flow between Merced and Lincoln (S15A Fig). This approach results in a conservative null model for selection because SNPs simulated under low migration rates between populations likely mimic SNPs that experience local selection. After calculating PBS values [13] from these simulated SNPs, we used the distribution to identify outliers above the simulated 99.9^th^ percentile (corresponding PBS: 0.252). There were 37,169 SNPs located in 6,913 genes above the 99.9^th^ percentile of the simulated distribution (red dashed line in S15 Fig). The ^Thr^755^Met^ *Epas1* SNP was highly significant (S15B Fig), as were the two linked noncoding SNPs (S15B Fig). These results provide strong evidence for spatially-varying selection on *Epas1* genotype and suggest that the associated phenotypic effects have fitness consequences in the wild.

**Fig 4:**
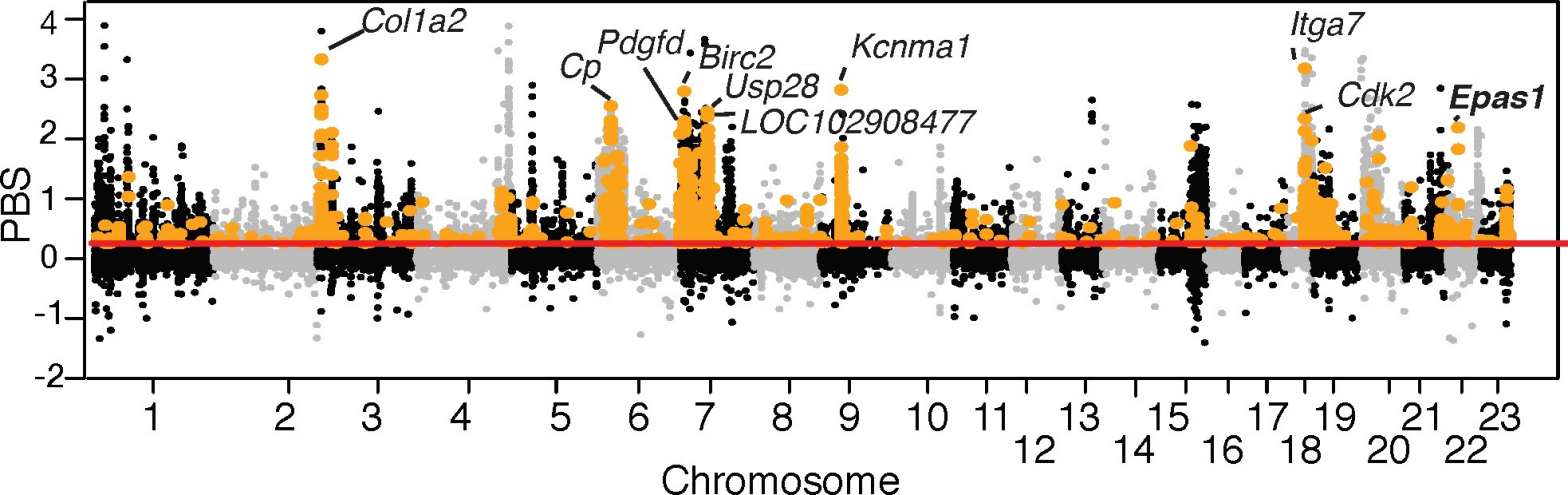
*Epas1* is an outlier in a demographically-controlled selection scan. **Exome-wide distribution of population branch statistic (PBS) for Mt. Evans mice based on 5,182,530 SNPs from the exome.** Horizontal line (red) indicates the top 0.1% percentile PBS of 500,000 neutral SNPs simulated under a realistic demographic scenario, and SNPs in hypoxia-related genes are highlighted in orange. The top ten hypoxia-related genes are labeled, with *Epas1* indicated in bold text. SNPs with a PBS value below the top 0.1% percentile have been randomly down-sampled (1:20) for ease of plotting.

Although we had a specific focus on the role of *Epas1* in physiological adaptation to high altitude, our demographically-corrected exome scan also identified a number of other promising candidates for high-altitude adaptation. Among the PBS outliers relative to the simulated null, 37 GO categories and 13 KEGG pathways were significantly enriched (FDR p-value <0.05; S10 Table). Many of these enriched categories are related to known physiological mechanisms for maintaining homeostasis under hypoxia, such as “response to oxygen-containing compound” (GO:1901700), “regulation of systemic arterial blood pressure” (GO:0003073), and “circulatory system process” (GO:0003013). We focused our examination on outlier SNPs that are located within 1,247 hypoxia-related genes identified by Zhang *et al*. [14] (S11 Table) that represent a set of candidates compiled from “hypoxia” and “hypoxia inducible factor” keyword searches in multiple sources [14]. Of the 6,913 genes with outlier SNPs, 353 genes overlapped with the set of 1,247 hypoxia-related genes from Zhang *et al*. [14] (S11 Table; S12 Table; Fig 4), representing a significant enrichment of hypoxia-related genes (one-sided Fisher's exact test; FDR-corrected p-value <0.001). *Epas1* was the tenth highest ranking gene (S12 Table).

The top three hypoxia-related genes were *Collagen type I alpha 2* (*Col1a2*) (highest-ranking SNP (PBS: 3.33), *Integrin subunit alpha 7* (*Itga7*) (PBS: 3.18) and *Potassium calcium-activated channel subfamily M alpha 1* (*Kcnma1*) (PBS: 2.82). *ColIa2* encodes one of the most abundant types of collagens that is a ubiquitous component of connective tissue in several organs, whereas *Itga7* and *Kcnma1* may both play roles in muscle structure and function. *Itga7* is expressed in skeletal and cardiac muscles, where it may play a developmental role [54], and *Kcnma1* encodes the pore-forming subunit of an ion channel that is integral to smooth muscle control [55]. Other high-ranking genes of note include *Ceruloplasmin* (*Cp*), which was the seventh highest ranking gene (PBS: 2.55; S12 Table) and is involved in iron transport across the cell membrane, in stimulation of erythropoiesis, and in nuclear translocation of HIF1 [56]. Further exploration of these hypoxia-related genes, as well as other targets of selection in high-altitude deer mice, is the focus of an ongoing study.

## Discussion

While many studies have identified hypoxia-inducible factors (HIFs) as candidates for high-altitude adaptation (e.g., [8,13–15,57,58]), few have experimentally documented phenotypic effects of mutations in these genes on aspects of systems-level physiology [18]. Yet, these types of genotype-to-phenotype association studies are necessary for identifying functional links that can advance multiple fields from evolutionary biology to biomedicine [59,60]. We combined a series of complimentary population genetic analyses and physiological experiments to test the role of HIFs in high-altitude adaptation of deer mice. We first identified a non-synonymous and polarity-altering polymorphism in the coding region of *Epas1* (which encodes HIF2α) that exhibited an extreme allele frequency difference between highland and lowland populations (Fig 1A). Moreover, the high-altitude *Epas1*^H^ allele, which is derived in the *P*. *maniculatus* sub-clade, varied predictably with altitude in mice sampled throughout the western United States (Fig 1B and 1C). These results suggested that variation at *Epas1* may contribute to well-characterized physiological differences observed between highland and lowland deer mice [25,26,31–39]. Physiological tests of this hypothesis demonstrated that *Epas1* polymorphism was not associated with many traits known to be regulated by HIFs [11]. Most notably we did not detect an association between *Epas1* genotype and hematocrit or hemoglobin concentration (S3 Table, S4 Table) unlike studies in high-altitude indigenous Tibetans [12], nor did we detect associations with adaptive variation in breathing pattern [34] or skeletal muscle fiber type [32] shown in high-altitude deer mice (see S5 and S6 Tables). If the observed mutation at *Epas1* does affect such traits, our results suggest that other mechanisms compensate for these effects in mice that have developed at high altitude.

Although we did not detect an effect of *Epas1* genetic variation on several HIF-related traits, we documented an unprecedented relationship between *Epas1* genotype and cardiovascular function. Experimental hypoxia exposures demonstrated that mice homozygous for *Epas1*^H^ maintain an elevated heart rate under physiologically and ecologically relevant levels of hypoxia (Fig 2). Moreover, *Epas1* genotype appears to have systematic effects on pathway gene expression, leading to downregulation of genes involved in catecholamine biosynthesis and secretion in the adrenal gland (Fig 3A and 3B), and upregulation of HIF targets in the left ventricle of the heart (Fig 3B and 3C). While the effects of *Epas1* genotype on the expression of any single gene are weak, the combined effects of subtle but concerted shifts in expression of genes in these candidate pathways were detectable and varied predictably among genotypes. Finally, formal demographically-controlled selection scans revealed that *Epas1*, and a number of other genes in hypoxia response pathways, have been a target of natural selection at high altitude (Fig 4). These results suggest that the associated phenotypic differences in cardiovascular function and gene regulation contribute to fitness differences in the wild.

This study provides the first documentation of a relationship between naturally occurring genetic variation at *Epas1* and heart rate under hypoxia. The maintenance of an elevated heart rate during hypoxia could be the direct phenotypic target of selection on *Epas1* in order to improve circulatory O_2_ delivery at high altitude. Assuming that there are not opposing differences in stroke volume between genotypes, increased heart rate should result in an increased cardiac output and thus enhanced O_2_ delivery to systemic tissues [10]. Indeed, mice that were homozygous for the highland *Epas1* genotype tended to have slightly larger ventricles (Table S4), which although not statistically significant, suggest that lower heart rates are not compensated for with increases in heart size (and potentially associated increases in stroke volume). Elevated heart rates during hypoxia could also be a secondary consequence of adaptive changes in other functions of the cardiovascular control system that are the direct targets of selection. One possible target of selection is catecholamine release from the adrenal medulla, which we have recently shown is attenuated in high-altitude deer mice [46]. The genetic basis for this attenuation is unknown, but the patterns of variation in expression for genes related to catecholamine synthesis and release suggest that genetic variation in *Epas1* may be involved. Catecholamine release in hypoxia normally acts to induce vasoconstriction and constrain blood flow to many peripheral tissues [61], so the attenuation of catecholamine release in highland mice may serve primarily to minimize this effect and improve tissue blood flow during hypoxia. However, hypoxic vasoconstriction could normally lead to feedback activation of the baroreflex, which would tend to reduce heart rate and oppose other factors that tend to stimulate heart rate in hypoxia (i.e., activation of sympathetic neurons innervating the heart). This potential secondary effect of hypoxic vasoconstriction would be minimized by an attenuation of adrenal catecholamine release, such that heart rate would rise more in response to hypoxia. Therefore, in this hypothetical example, the primary target of selection (improvement in regional tissue blood flows via reductions in catecholamine release) has secondary consequences on the heart rate response to hypoxia.

Whatever the mechanism, the observed effects of *Epas1* genotype within highland mice mirror genetically-based population differences in heart rate between highlanders and lowlanders [34], suggesting that evolved population differences may result from allelic variation at *Epas1*. Humans native to the Qinghai-Tibetan plateau (2200–5200 m a.s.l) reach higher maximal cardiac outputs during exercise compared to closely related lowland Han Chinese when tested at high altitude [62]. These results suggest that changes in cardiovascular function or control–either at rest, as in deer mice, or during exercise, as in Tibetan humans–may be a common adaptation to high altitude. The potential benefit to circulatory O_2_ delivery conferred by these cardiovascular changes is likely one of many adaptations that improve metabolic homeostasis across systemic tissues in highlanders. These other potential adaptations include evolved changes in breathing and respiratory gas exchange [34], higher hemoglobin O_2_ affinity [28], enhanced capacity for O_2_ diffusion into metabolically active muscles [26,32,37], as well as changes in the density, intracellular distribution, and function of muscle mitochondria [37,38]. All of these modifications are likely to contribute to an enhancement of aerobic performance under O_2_ deprivation [24,25,32].

The results of this study demonstrate a role for coding changes in a transcription factor in environmental adaptation. The majority of the systems-level traits we measured (e.g., breathing, metabolism) were remarkably similar between *Epas1* genotypes, as were the transcriptome-wide effects of *Epas1* genotype on gene expression in the adrenal gland and heart, suggesting that the *Epas1* amino acid mutation has highly targeted phenotypic effects. Our results suggest that selection on high-level transcription factors such as *Epas1* contribute to local adaptation, so any negative pleiotropic effects associated with the evolved changes must be compensated by other mechanisms.

## Materials and methods

### Ethics statement

We handled and sampled mice in accordance with the University of Montana's Institutional Animal Care and Use Committee (AUP 029–16), the Colorado Department of Fish and Wildlife (License Number: 17TR2168a) and the United States Forest Service (Authorization ID: CLC772). Following protocol, mice were euthanized with an isofluorane overdose followed by decapitation.

### Exome design, capture, and high-throughput sequencing

To identify all annotated exons, we downloaded the *Peromyscus maniculatus bairdii* GFF v101 from NCBI (Accession GCF_000500354.1), and extracted all features annotated as an exon. The final set of unique, non-pseudogenized exonic regions consisted of 218,065 exons in 25,246 genes. A custom Roche NimbleGen SeqCap EZ Library kit capture a total of 226,973 regions (77,559,614 bp).

We extracted DNA from tissues of 85 deer mice (Lincoln, NE: n = 37; Mount Evans, CO: n = 48) and sheared DNA to ~300 bp using a Covaris E220 Focused Ultrasonicator. Genomic libraries for each individual were prepared using 200 ng of sheared DNA with a NEBNext UltraII kit and unique index following manufacturer’s protocols. We pooled batches of 24 indexed libraries prior to target enrichment and PCR amplification following the NimbleGen Seq Cap EZ protocol (Roche). Quality control for each capture pool included a check of size distribution and a check for enrichment of targeted regions and no enrichment of non-targeted regions using qPCR. Each capture pool of 24 individuals was sequenced with 100 bp paired-end sequencing on an Illumina HiSeq 4000. We extracted and quantified DNA samples for the California deer mice (Merced, CA; n = 15) at the Museum of Vertebrate Zoology, UC Berkeley, before shearing one μg of genomic DNA to less than 500 bp with a Biorupter (Diagenode). We prepared barcoded Illumina sequencing libraries using the Meyer and Kircher [63] protocol, then amplified libraries with Phusion High-Fidelity DNA Polymerase (Thermo Scientific) for 6–8 cycles during the indexing PCR. Exome enrichment was conducted with a custom capture design from the SeqCap EZ Developer Libary (Nimblegen) that was almost identical to that used in the 85 non-CA samples. Captures were quantified, pooled proportionally to the amount of DNA in each, and sequenced using 100bp pair-end sequencing on an Illumina HiSeq4000.

Data pre-processing and variant discovery on all samples followed the recommendations of the Broad Institute GATK v3.7-0-gcfedb67 Best Practices pipeline. We trimmed reads of adapter sequences and for a minimum base quality of 20 using *fastq_illumina_filter 0*.*1* (https://mcbl.readthedocs.io/en/latest/mcbl-tutorials-PF-clean.html) and *trim_galore 0*.*3*.*1* (http://www.bioinformatics.babraham.ac.uk/projects/trim_galore/). We used *bwa mem* [64] to align and map forward and reverse reads to the *Peromyscus maniculatus baiardii* genome. We removed duplicates using *samtools rmdup* [65], then added read group information using *picard*. (http://picard.sourceforge.net). To generate a set of “known” variant sites for *GATK Base Quality Score Recalibration (BQSR)*, we genotyped each individual using *samtools mpileup* (-q 30 -Q 30) and *bcftools call*, then filtered genotypes to have a minimum depth of coverage (minDP) of 10 and minimum genotype quality (minGQ) of 30, and only used those variants observed in at least two individuals. The resulting set of variant positions was used with the -*knownSites* flag during *GATK BQSR*. A subsequent round of *BQSR* was completed and convergence of quality scores was verified using *GATK AnalyzeCovariates*. To genotype each sample, we used *GATK HaplotypeCaller* with the ‘--emitRefConfidence' flag, then called variants *GATK GenotypeGVCFs*. We combined GVCFs and filtered them to remove SNPs with a quality of depth <2.0, a FS > 60, mapping quality < 40, mapping quality rank sum < -12.5, and read position rank sum < -8.0. We implemented all processing steps in *GATK* using the ‘--interval' flag, a bed file of capture regions, and a ‘--interval_padding’ of 200 bp. These processing steps resulted in a total of 106,883,914 sites among all individuals.

After assessing the quality of filtered reads using the *vcftools* package[66], we further filtered variants so that a site was called in at least 50% of individuals, was bi-allelic, and each site had a minDP of 5 and minGQ of 20. We proceeded with a set of 5,183,434 high-quality bi-allelic variants, with a mean depth of coverage of 18.10±6.38 X (S1 Fig).

### Population genetic structure

We assessed population genetic structure of Mount Evans, Lincoln, and Merced mice using principal components analysis within PLINK [40] and Admixture [41]. Prior to running the analyses, we pruned the set of variants to only sites with no missing data, and not linked (using the “--indep-pairwise 50 5 0.5” option within PLINK). The final set of variants for these analyses consisted of 296,196 bi-allelic sites.

### Allele frequency variation in HIF genes

In order to examine altitudinal patterns of allele frequency variation of the genes encoding HIF-1α, HIF-2α, and HIF-3α, we calculated pairwise F_ST_ using vcftools, then the Population Branch Statistic (PBS) [13] for each of the 5,183,434 bi-allelic variants. Using the ‘ecdf’ function in R, we used the empirical distribution of PBS values and set a threshold of 99.9% for significance (corresponding PBS: 0.886). We used Ensembl’s Variant Effect Predictor [67] with the *P*. *maniculatus* reference genome annotation data set to identify SNPs located in HIF genes. For the three outlier SNPs located in *Epas1*, we calculated pairwise linkage between each SNP as the squared correlation coefficient, r^2^, using the ‘--geno-r2’ function within vcftools [66],

### Geographic and phylogenetic survey

To fully assess the geographic and phylogenetic extent of variation in *Epas1*, we genotyped 266 *P*. *maniculatus* samples from across the western US (S1 Table), plus samples of *P*. *keeni*, *P*. *melanotis*, *P*. *hylocytes*, *P*. *attwateri*, *P*. *melanophrys*, *P*. *eremicus*, *P*. *polionotus*, *P*. *leucopus*, *Reithrodontomys montanus*, and *Phyllotis xanthopygus* (S2 Table). We obtained tissue samples from our existing freezer collections or museums (Museum of Southwestern Biology at the University of New Mexico, or Museum of Comparative Zoology, Harvard University). From each sample, we extracted DNA then PCR amplified *Epas1* with custom exonic primers (“epas1_snp_L” and “epas1_snp_R”; S13 Table) designed from the *P*. *maniculatus bairdii* genome under the following conditions: 94°C for 2 mins; 30 cycles of 94°C for 45 sec, 58°C for 1 min, 72°C for 1 min; then 72°C for 10 mins. To improve amplification specificity for *P*. *maniculatus* samples, we used modified primers and PCR conditions (“epas1_set1_F” and “epas1_set1_R”; S13 Table): 94°C for 2 mins; 35 cycles of 94°C for 30 sec, 62°C for 30 sec, 68°C for 1 min; then 68°C for 10 mins. Technicians at Genewiz (South Plainfield, NJ) cleaned amplified products and sequenced them in both directions. We called genotypes after aligning sequences to the reference sequence using Geneious 8.1.8. We calculated population allele frequencies within each sampling locality, and obtained elevation for each locality from GPS data recorded upon sampling, or from Google Maps. We mapped these allele frequency data on a map of elevation (data downloaded from www.worldclim.org) using the ‘maps’ package in R. Finally, we placed *Epas1* genotype for each sequenced species on a *Peromyscus* phylogeny constructed previously [68–70].

### Cline analysis for Epas1 and Hemoglobin genes

We tested whether the *Epas1* allele frequencies follow a clinal pattern using the R package HZAR [71]. In HZAR, genetic data are fit to equilibrium cline models using the Metropolis–Hastings Markov chain Monte Carlo (MCMC) algorithm, and parameters such as the cline center (*c*) and width (*w*) are estimated. *c* and *w* characterize the location within the transect where the variable changes most rapidly, and the values of these parameters can be estimated within HZAR by 15 models that differently estimate the exponential decay on either side of the cline center, as well as the minimum or maximum frequencies. We used as input the *Epas1* allele frequency data and elevation for populations of deer mice sampled across the Rocky Mountain to Great Plains elevational cline, and used a burn-in of 10000. We compared the *Epas1* cline to that for previously published hemoglobin haplotype frequencies in deer mice [43].

### Physiological effects of allelic variation at *Epas1*

To test for physiological effects of allelic variation at *Epas1*, we captured deer mice from a single interbreeding population in which the high-altitude allele is segregating. We collected adult deer mice from the summit of Mt. Evans (Clear Creek Co., Colorado, USA; 39°35’18” N, 105°38’38” W; 4,350 m above sea level; PO_2_ ~ 95.6 mm Hg) in August 2016 and 2017 and screened the *Epas1* allele (a C/T polymorphism at nucleotide position 2264, hereafter ^C^2264^T^). From each individual, we sampled an ear clip sample, extracted DNA, then genotyped *Epas1* ^C^2264^T^ using a custom restriction enzyme digest assay. Briefly, we PCR amplified *Epas1* with custom exonic primers (S13 Table) designed from the *P*. *maniculatus bairdii* genome and amplified under the PCR conditions specified above. For all amplified PCR products, we cut *Epas1* at ^C^2264^T^ by incubating the PCR product with the BsaHI restriction enzyme at 37°C for 1 hour followed by a heat denaturation for 20 mins. We called *Epas1* genotypes via gel electrophoresis (T ~675 bp; C ~300 bp;), then subsequently confirmed field genotypes with Sanger sequencing at Genewiz.

### Acute hypoxia responses with pulse oximetry

At the University of Denver Mt. Evans field station (3230 m a.s.l.; ~15kPa O_2_), we screened these Mt. Evans mice with alternative *Epas1* genotypes for a suite of physiological responses involved in O_2_ transport and utilization and/or known to be influenced by HIFs. We measured hypoxia responses in mice (26 *Epas1*^H/H^, 13 *Epas1*^H/L^, and 4 *Epas1*^L/L^) using previously described barometric plethysmography, respirometry, and pulse oximetry techniques [34,72]. We placed each mouse in a whole-body plethysmograph (chamber volume: 530 ml) that was supplied with hyperoxic air, mixed to simulate the partial pressure of O_2_ (PO_2_) at sea level (21 kPa O_2_, balance N_2_), at a rate of 600 ml min^-1^. We gave mice 20–60 min to adjust to the chamber and stabilize their breathing and metabolism. We recorded measurements for an additional 20 min at 21 kPa O_2_, then exposed mice to 20 min stepwise reductions in inspired PO_2_ of 12, 10, 8, and 6 kPa. We set the incurrent gas composition by mixing dry compressed gases using precision flow meters and a mass flow controller, such that the desired PO_2_ was delivered to the chamber at a constant rate of 600 ml min^-1^. At the end of the experiment, we measured body temperature (T_b_) using a mouse rectal probe. We also measured T_b_ exactly 24 h later to determine resting T_b_ (this was used as a proxy for the resting T_b_ at the start of the experiment, which was not measured to prevent stress to the animal).

We determined breathing and O_2_ consumption rate (VO_2_) during the last 10 min at each PO_2_ by subsampling incurrent and excurrent airflows at 200 ml min^-1^. For incurrent and excurrent air, we measured water vapor (RH-300, Sable Systems) using a thin-film capacitive water vapor analyzer, then dried air with pre-baked drierite, and measured continuously for O_2_ and CO_2_ fraction using a galvanic fuel cell O_2_ analyzer and infrared CO_2_ analyzer (FOXBOX, Sable Systems). We used these data to calculate VO_2_ and CO_2_ production rate (VCO_2_), expressed at standard temperature and pressure (STP), using appropriate equations for dry air as described by Lighton [73]. We measured breathing frequency and tidal volume from changes in flow across a pneumotachograph in the plethysmograph wall, detected using a differential pressure transducer. We calculated tidal volume using established equations [75,76] and assuming a constant rate of decline in T_b_ with declining PO_2_, which we have previously shown results in similar tidal volumes to those calculated using direct T_b_ measurements at each PO_2_ [72], and is expressed at STP. We calculated the following parameters: total ventilation (the product of breathing frequency and tidal volume), ventilatory equivalent for O_2_ (total ventilation divided by VO_2_), and percent pulmonary O_2_ extraction (VO_2_ divided by the product of total ventilation and inspired O_2_ fraction). We measured SaO_2_ and heart rate using the MouseOx Plus pulse oximeter collar sensors and associated software (Starr Life Sciences, Oakmont, PA, USA). Use of the collars was enabled by removing a small amount of fur from the skin around the neck one day before experiments.

We tested for effects of *Epas1* genotype on cold-induced VO_2_ max (thermogenic capacity), an ecologically relevant measure of whole-organism aerobic performance for which there is an evolved difference between lowland and highland deer mice [24]. To do this we measured maximum rates of oxygen consumption in a hypoxic, heliox atmosphere (21% oxygen, 79% helium) using open-flow respirometry. All trials were conducted at the summit of Mt. Evans. The use of heliox ensures that VO_2_ max can be measured without risking cold injury, since rates of heat loss in heliox are several times greater than that of ambient air [74]. For each trial, we equilibrated heliox gas mixtures with atmospheric pressure of the Mt. Evans summit (12 kPa). Mass flow controllers helped pump the heliox mixture into copper coils inside a temperature control chamber. The cooled gas was pumped into an animal chamber and an empty baseline chamber at a rate of ~750 ml min^-1^. Excurrent air from the animal and baseline chambers was sampled at a rate of ~130 ml min^-1^, dried with magnesium perchlorate and scrubbed of CO_2_, redried with drierite, and passed through an oxygen analyzer. We defined thermogenic capacity as the maximum VO_2_ averaged over a continuous 5-min period. We tested for the influence of genotype on thermogenic capacity using an analysis of covariance (ANCOVA) with body mass as covariate.

### Tissue and organ sampling and phenotyping

Mice recovered for 2–3 days after the hypoxia response experiments described above. We exposed recovered mice to 2 hours of deep hypoxia (6 kPa O_2_) and euthanized them with an isofluorane overdose followed by decapitation. We collected blood samples for hematocrit (in heparinized capillary tubes, spun for 5 minutes) and hemoglobin content (Hemocue, Sweden). We dissected the heart, then isolated and weighed the ventricles before freezing them separately in liquid N_2_. We determined lung volume by volumetry [75]. We weighed and froze in liquid N_2_ one gastrocnemius muscle, then coated the other in embedding medium and froze it in liquid N_2_-cooled isopentane for histology. We froze other organs directly in liquid N_2_.

We measured muscle capillarity using histological methods in a subset of the mice (17 *Epas1*^H/H^, 13 *Epas1*^H/L^, and 4 *Epas1*^L/L^) chosen to ensure a balanced sex distribution (11 *Epas1*^H/H^ males, 6 *Epas1*^H/H^ females, 11 *Epas1*^L/-^ males, 6 *Epas1*^L/-^ females) and body mass (21.49 ± 4.05 g for *Epas1*^H/H^, 21.76 ± 5.43 g for *Epas1*^H/L^, 21.01 ± 4.12 g for *Epas1*^L/L^; means ± SEM) between the three groups. We prepared full transverse sections of the gastrocnemius muscle as previously described [32,76]. Briefly, after cutting 10 μm tissue sections transverse to the muscle fiber length using a cryostat, we identified capillaries by staining samples for alkaline phosphatase activity following previous studies [32,76]. We used bright-field microscopy to systematically collect images from across the entire gastrocnemius, and used ImageJ software [77] to count the number of capillaries and muscle fiber in each image and measured capillary density, average number of capillaries per muscle fiber, and average transverse area of muscle fibers. We used NIS-Elements D Imaging Software (v. 4.30, Nikon Instruments) to measure the number, perimeter, and capillary surface densities of individual capillaries within each image. We determined a sufficient number of images to analyze to account for heterogeneity across the gastrocnemius, determined by the number of replicates necessary to yield a stable mean value, following ref. [32]. This required analysis of roughly half of the entire section, with images spread evenly across the section, which was found to be more than sufficient to accurately represent average values across the entire muscle. For all histological measurements, the observer was blind to genotype during analysis.

We used the remaining gastrocnemius muscle tissue in metabolic enzyme assays that we have previously described [76]. After removing embedding medium from the muscle tissues we powdered samples under liquid N_2_ and homogenized them in ice-cold homogenization buffer [76]. We centrifuged homogenates at 1000*g* for 1 min at 4°C, discarded the pellet, and stored the homogenate on ice until assay. We assayed activities of cytochrome c oxidase (COX), citrate synthase (CS), and lactate dehydrogenase (LDH) in triplicate at 37°C using a 96-well microplate reader. Assay conditions in mM were as follows: COX, 100 KH_2_PO_4_, 0.2 reduced cytochrome c*, pH 8.0; CS, 100 KH_2_PO_4_, 0.5 oxaloacetate*, 0.15 acetyl-coA, 0.15 5,5′-dithiobis-2-nitrobenzoic acid (DTNB), pH 8.0; LDH, 100 KH_2_PO_4_, 0.15 NADH, 2.5 pyruvate*, pH 7.2. We determined maximal activities by measuring the change in absorbance over time at 550 nm for COX (ε = 28.5 mM^-1^ cm^-1^), 412 nm for CS (ε = 14.15 mM^-1^ cm^-1^), and 340 nm for LDH (ε = 6.22 mM^-1^ cm^-1^), and subtracting the background rate from the rates measured in the presence of all substrates.

### Statistical analyses of physiological effects of allelic variation at *Epas1*

To assess the influence of *Epas1* genotype on physiology and tissue/organ phenotypes, we used linear mixed effect models and included body mass, genotype, and acute PO_2_ (when appropriate) as fixed effects. We initially included year (2016 or 2017) and sex as random effects, but removed them from all models because they were never found to be significant (P>0.25). We removed body mass from models in which its effect was not significant for variables that we did not have any *a priori* expectation of allometric scaling (heart rate, SaO_2_, hematology, and gastrocneumius muscle capillarity and enzyme activities). We conducted Holm-Sidak pairwise post-tests on significant models, and used R (v. 3.4.3) and the *lme4* package for all statistical analysis, with a significance level of 0.05. We report VO_2_, total ventilation, and tidal volume relative to body mass to enable comparison to the literature, but we used the absolute data (i.e., not expressed relative to body mass) for statistical analyses as described above.

### Transcriptomic analysis of differential gene expression

We used high throughput sequencing to test for effects of *Epas1* genotype on gene expression in adrenal gland (8 *Epas1*^H/H^; 8 *Epas1*^H/L^; 3 *Epas1*^L/L^) and heart tissue (7 *Epas1*^H/H^; 9 *Epas1*^H/L^; 3 *Epas1*^L/L^). We chose the adrenal gland because of its role in stimulating heart rate *via* catecholamine release. We assayed gene expression using TagSeq, a 3’ tag-based sequencing following ref. [45]. We extracted RNA from 25 mg of tissue using TRI Reagent (Sigma-Aldrich), then assessed RNA quality using TapeStation (Agilent Technologies; RIN > 7). The Genome Sequencing and Analysis Facility at the University of Texas at Austin prepared TagSeq libraries, which were sequenced using Illumina HiSeq 2500. Sequencing generated an average of 4.6M reads per individual. We processed raw reads following Lohman et al. [45] and mapped them to the *P*. *maniculatus genome* using bwa [64]. We used *featureCounts* [78] to generate a table of transcript abundances. Since genes with low read counts are subject to increased measurement error [79], we excluded those with less than an average of 10 normalized reads per individual using the *filterByExpr* function in *edgeR*. We retained a total of 12,237 and 10,509 genes after filtering for adrenal and heart transcriptomes, respectively.

We used two complementary approaches to compare levels of transcript abundance among *Epas1* genotypes: (1) A whole-transcriptome differential expression analysis was conducted to identify genes that were differentially expressed in each tissue. (2) We performed candidate differential expression analysis on two *a priori* gene sets aimed at testing whether genes related to the HIF cascade and/or catecholamine synthesis/transport exhibited concerted changes in gene expression among alternative genotypes. We conducted the whole-transcriptome differential expression analysis in *edgeR* [80]. The function *calcNormFactors* was used to normalize read counts among all libraries, after model dispersion was estimated for each transcript separately using the function *estimateDisp* [81]. We tested for differences in transcript abundance by first fitting a quasi-likelihood negative binomial generalized linear model to raw count data (*glmQLFit* function), which included a single main effect of genotype (*Epas1*^L/L^ was used as a reference for comparing against *Epas1*^H/L^ and *Epas1*^H/H^). P-values were calculated using a quasi-likelihood F test using the *glmQLFTest* function. We controlled for multiple testing by enforcing a genome-wide false discovery rate correction of 0.05 [82]. We identified candidate HIF target genes from the literature [83,84] and Kyoto Encyclopedia of Genes and Genomes database [85] as those with known function in HIF signaling, and those that have an unknown function but contain HIF binding sites [84]. We ascertained catecholamine-related genes based on annotation in the Gene Ontology (GO) database in AmiGO (amigo.geneontology.org). A total of 277 HIF targets and 149 catecholamine related genes (S7 Table) were identified.

To determine whether there were concerted shifts in gene expression for the candidate gene sets among genotypes, we calculated log fold-change in expression between *Epas1*^L/L^ mice and mice heterozygous and homozygous for the high-altitude allele in *edgeR*. The distribution of fold-change values between candidate gene sets and the transcriptome-wide background (candidate genes excluded) was compared using Kolmogorov-Smirnov (K-S) tests using the function *ks*.*test* in R. We then conducted a randomization procedure to test whether the observed D-statistic for K-S tests was greater than a null distribution; the null distribution of D-statistic values was produced by 1000 random draws of gene sets that were of equal size to candidate sets (adrenal: HIF: *n* = 207; catecholamine: *n* = 79; left ventricle: HIF: *n* = 207; catecholamine: *n* = 55) and comparing those to the transcriptome-wide background.

### Demographic modeling and null PBS distribution

We estimated the demographic history of highland and lowland deer mice using synonymous SNPs in *∂a∂i* [50]. We filtered SNPs in Hardy-Weinberg equilibrium (*p*<0.001) and excluded sites with >25% missing data per population using vcftools, resulting in 287,336 SNPs to generate a folded site-frequency spectrum. We then estimated effective population sizes (*N*_*e*_), divergence times (*T*), and pairwise migration rates (*m*) between highland deer mice from Mt. Evans, CO (*n* = 48) and deer mice from Lincoln, NE (*n* = 37) and Merced, CA (*n* = 15). We assumed that any migration between Lincoln and Merced populations would occur indirectly through the central Mt. Evans population and thus we did not perform a pairwise demographic analysis for Lincoln and Merced. For our pairwise population comparisons (Mt. Evans-Lincoln and Mt. Evans-Merced), we calculated maximum-likelihood (ML) parameters for demographic models with and without a single symmetrical migration parameter and with an effective population size parameter (*u*; proportional change in *N*_*e*_ relative to the ancestral population immediately following the split). We observed that maximum likelihood parameters under models of no migration or a single symmetrical migration rate strongly underestimated the relative abundance of highly differentiated SNPS, resulting in poor fit to the empirical 2D-SFS (S14 Fig). We also tested a model which included heterogeneous migration rates among loci in the genome. Here, we included two symmetrical migration rates, one for proportion *P* of SNPs and one for proportion 1-*P* of SNPs, where we also estimate the *P* parameter. For each demographic model, we performed 25 independent runs with starting parameter values sampled randomly from a uniform prior distribution (0<2*N*_*e*_*m*<10; 0.01<2*N*_*e*_*t*<10; 0.1<*u*<10; 0.5<*P*<1.0). We selected the optimal demographic model based on an adjusted likelihood ratio test using the Godambe Information Matrix [52]. We estimated 95% confidence intervals for population size, divergence time, and migration rate parameters using the Godambe Information Matrix with 100 replicate bootstrap data sets consisting of randomly sampled SNPs spaced at least 10 kb apart (14250 SNPs for each data set). We calibrated theta estimates based on the ratio of all callable sites to SNPs under the same filtering regime and assuming a mutation rate of 5.4x10^-9^ per base per generation (house mouse; ref. [86]).

To establish a null PBS distribution, we simulated 500,000 neutral SNPs across three populations in *msms* [53] under our estimated demographic model. Given our optimal models included two symmetrical migration rate parameters applied to different sets of SNPs we simulated proportion *P* of SNPs under high migration rates (85%) and proportion 1-*P* of SNPs under low migration rates (15%) and combined the two simulated data sets. We used msstats (https://github.com/molpopgen/msstats) to obtain *F*_ST_ values for SNPs between each population and calculated PBS based on the equation in Yi et al. [13].

### Demographically corrected exome scan with the Population branch statistic

We used the simulated distribution of PBS values, and set a significance threshold of 99.9% (corresponding PBS_sim_: 0.199). We focused our examination on outlier SNPs that are located within 1,247 hypoxia-related genes from Zhang *et al*. [14] (S11 Table). The genes from Zhang *et al*. represent a set of candidates compiled from “hypoxia” and “hypoxia inducible factor” keyword searches in multiple sources, including the UCSC Genome Browser, Ensembl, NCBI, UniProt, and RefSeq. For each *P*. *maniculatus* gene containing outlier SNPs, we found the corresponding *Mus musculus* gene, then used gProfiler [87] to identify enriched gene ontology categories above a false discovery rate corrected significance of 0.05, using strong hierarchical filtering.

## Supporting information

S1 Text File containing supplemental results for population genetic, cline, and physiological analyses. (DOCX)

S1 Table Sampling locations and frequency of Epas1 alleles for 266 *Peromyscus maniculatus* samples used to generate map and cline in Fig 1. Sampling locations in bold text were used to generate cline in Fig 1C.
(XLSX)

S2 Table Sampling locations, museum accessing numbers, and *Epas1* genotypes for *Peromyscus* and broader phylogenetic sampling. (XLSX)

S3 Table Blood characteristics and organ masses of *Epas1* variants of deer mice. Values are expressed as mean ± SEM; n = 31 for *Epas1*^H/H^, n = 13 for *Epas1*^H/L^, n = 4 for *Epas1*^L/L^ variants.
(XLSX)

S4 Table F- and P-values for mixed linear effect models of blood and organ mass variables in *Epas1* variants of deer mice. n.s. not significant and excluded from the final model.
(XLSX)

S5 Table F- and P-values from mixed linear models of gastrocnemius muscle capillarity and enzyme activity in *Epas1* variants of deer mice. n.s. not significant and excluded from the final model.
(XLSX)

S6 Table F- and P-values from mixed linear models of ventilatory and metabolic responses of *Epas1* variants of deer mice. n.s. not significant and excluded from the final model.
(XLSX)

S7 Table Candidate set of hypoxia inducible factor (HIF) cascade target genes and catecholamine synthesis and secretion genes used in targeted differential gene expression analysis. Candidate genes were curated from the literature (Ortiz-Barahona et al. 2010; Dengler et al. 2016) and publically available databases (KEGG: Kyoto Encyclopedia of Genes and Genomes; AmiGO).
(XLSX)

S8 Table Results of differential gene expression among candidate HIF and catecholamine genes. Table reports average log_2_ fold-change (logFC) in expression, average log2 read counts per million (logCPM), and the F-value (F) from quasi-likelihood F tests, p-value, and false-discovery rate corrected p-value (FDR) of quasi-likelihood generalized linear models comparing expression between *Epas1*^L/L^ vs. *Epas1*^H/H^ and *Epas1*^H/L^ for adrenal and left ventricle tissues. Gene names are *Mus musculus* gene ids.
(XLSX)

S9 Table Maximum likelihood parameter estimates and 95% confidence intervals (CI) for demographic model. (XLSX)

S10 Table Results from gProfiler for gene ontology enrichment of genes containing SNPs above the 99.9^th^ percentile of the empirical distribution of PBS values. Only categories significant above a Benjamini-Hochberg false-discovery rate of 0.05 are included.
(XLSX)

S11 Table The list of hypoxia-related genes used in this study. The list of genes was extracted from Zhang et al. (2014), then orthologs in *Mus musculus* and *Peromyscus maniculatus* were identified using DAVID and custom scripts.
(XLSX)

S12 Table Hypoxia-related genes containing SNPs with PBS values above the 99.9^th^ percentile of the empirical distribution. For each gene, the PBS value, genomic location, and percentile of the highest-ranking SNP is provided.
(XLSX)

S13 Table *Epas1* primer sequences. Primers were used to amplify *Epas1* prior to restriction enzyme digest for screening of alternative alleles. Sequences are given 5' to 3'. See supplemental text for PCR reaction conditions.
(XLSX)

S1 Fig Mean Depth of Coverage for 100 Exomes. Distribution of mean depth of coverage for 100 exome samples used in this study. The final set of quality-filtered sites consists of 5,182,530 high-quality bi-allelic variants sequenced at approximately 18X coverage. Red dashed vertical line indicates the mean.
(PDF)

S2 Fig PCA of Three Focal Populations. Principal components analysis of Mount Evans (n = 48), Lincoln (n = 37), and California (n = 15) mice, based on genotypes from 296,196 exome-wide LD-pruned SNPs with no missing data.
(PDF)

S3 Fig Population assignment made via *Admixture* for K = 2 to K = 3 for 100 individuals. A) *Admixture* was run on a set of 296,196 exome-wide LD-pruned SNPs with no missing data. Each vertical bar represents an individual, with the colors corresponding to proportion assignment at each value of K. B) The lowest cross validation error rate was at K = 1; however, higher values of K are biologically meaningful and are therefore shown here.
(PDF)

S4 Fig Density distribution of population branch statistic (PBS) values calculated for Mount Evans, using Lincoln and Merced populations as outgroups. The mean (green vertical dashed line), 99^th^ (blue vertical dotted line), and 99.9^th^ (red dash-dotted line) values of the empirical distribution are shown. Orange vertical lines indicate three outlier SNPs located in *Epas1*, with the rightmost line indicating the ^Thr^755^Met^ SNP.
(PDF)

S5 Fig PBS Values for exonic SNPs in *Epas1*. Manhattan plot of PBS values for all SNPs (black dots) located within all exons of *Epas1*. Exome-wide values for mean, 99%, and 99.9% percentile PBS values are shown, and three outlier SNPs above the 99.9^th^ percentile located in *Epas1* are highlighted in orange. Pairwise linkage disequilibrium estimates (measured with the squared correlation coefficient, r^2^) for each SNP pair are provided.
(PDF)

S6 Fig Correlation between *Epas1*^*H*^ frequency and sampling elevation. Significant positive correlation of high-elevation allele frequency with sampled elevation, based on genotyping 23 populations.
(PDF)

S7 Fig Clinal variation in two-locus HBB haplotype frequencies. Clinal variation for nine *P*. *maniculatus* populations sampled along a 4500 m altitudinal cline from the Great Plains of Nebraska to the Rocky Mountains in Colorado. Data from Storz et al. 2012 *Genetics*.
(PDF)

S8 Fig Histological analysis of capillarity in the gastrocnemius muscle. Capillaries were identified by staining for alkaline phosphatase activity. The oxidative core (A,C,E) and the outer less oxidative region (B,D,F) of the muscle is shown for representative individuals possessing *Epas1*^H/H^ (A,B), *Epas1*^H/L^ (C,D), and *Epas1*^L/L^ (E,F) genotypes. All images are shown at the same scale, and the scale bar represents 100 μm.
(PDF)

S9 Fig Statistical analysis of capillarity in the gastrocnemius muscle. There were no differences in capillarity in the gastrocnemius muscle between deer mice with different *Epas1* genotypes. Capillarity was quantified using the following measurements: A) capillaries per muscle fiber, B) capillary surface density, C) capillary density, and D) transverse muscle area per muscle fiber. Sample sizes: n = 16 *Epas1*^H/H^, n = 13 *Epas1*^H/L^, and n = 4 *Epas1*^L/L^ variants.
(PDF)

S10 Fig Activity of oxidative enzymes in the gastrocnemius muscle. The activities of oxidative enzymes, i.e. A) cytochrome c oxidase (COX) and B) citrate synthase (CS) in the gastrocnemius muscle were similar between deer mice with different *Epas1* genotypes, but C) lactate dehydrogenase (LDH) activity appeared to be lower in mice that were homozygous for the highland *Epas1* variant. † Significant difference in a post-hoc comparison between only *Epas1*^H/H^ and *Epas1*^H/L^ genotypes. n = 16 *Epas1*^H/H^, n = 13 *Epas1*^H/L^, and n = 4 *Epas1*^L/L^ variants.
(PDF)

S11 Fig Ventilatory response of deer mice with varying *Epas1* genotype. Deer mice with different *Epas1* genotypes exhibited similar ventilatory responses to increasingly severe levels of acute hypoxia. Ventilation was quantified via A) total ventilation, B) arterial oxygen saturation, C) breathing frequency, and D) tidal volume. Sample sizes: n = 26 *Epas1*^H/H^, n = 13 *Epas1*^H/L^, and n = 4 *Epas1*^L/L^ variants.
(PDF)

S12 Fig Response of O_2_ consumption rate and body temperature under acute hypoxia. Deer mice with different *Epas1* genotypes exhibited similar declines in A) O_2_ consumption rate and B) body temperature in response to increasingly severe levels of acute hypoxia, and similar increases in C) ventilatory equivalent for O_2_ and D) pulmonary O_2_ extraction. Sample sizes: n = 26 *Epas1*^H/H^, n = 13 *Epas1*^H/L^, and n = 4 *Epas1*^L/L^ variants.
(PDF)

S13 Fig Heart rate response according to *Epas1* genotype. Deer mice that were homozygous for the highland *Epas1* variant exhibited a significantly greater increase in heart rate from normoxia (21 kPa O_2_) to environmentally realistic levels of hypoxia at 4300 m elevation (12 kPa O_2_). Measurements were made using a MouseOx Plus collar. * A significant pairwise difference between *Epas1*^H/H^ and *Epas1*^H/L^ mice. n = 26 *Epas1*^H/H^, n = 13 *Epas1*^H/L^, and n = 4 *Epas1*^L/L^ variants.
(PDF)

S14 Fig Results of demographic modeling in deer mice. The folded 2-dimensional site frequency spectra (2d-SFS) for deer mice from (A) Mt. Evans, CO, and Merced, CA, and (B) Mt. Evans, CO, and Lincoln, NE. For each pair of populations, we show the empirical 2d-SFS from whole exome data and the maximum likelihood 2d-SFS for demographic models with no migration, one migration rate, and two migration rates. Residuals reflect the overall fit of the model to the empirical data, where red indicates an overestimation of the number of SNPs by the model and blue reflects an underestimation.
(PDF)

S15 Fig Density distributions of PBS values under simulated and empirical models. **A)** Density plot of population branch statistic (PBS) values calculated for Mount Evans, using Lincoln and Merced populations as outgroups, from 10,000 SNPs simulated under modeled demography. Values for the mean (green vertical dashed line), 99^th^ (blue vertical dotted line), and 99.9^th^ (red dash-dotted line) percentiles of the simulated distribution are shown. B) Density plot of empirical PBS values (same as in S4 Fig), but with significance thresholds based on simulated 99^th^ and 99.9^th^ percentile shown.
(PDF)
