# Supplementary material for: Physiological and genomic evidence that selection on the transcription factor Epas1 has altered cardiovascular function in high-altitude deer mice
Source: PLoS Genet. 2019 Nov 7;15(11):e1008420. doi: 10.1371/journal.pgen.1008420 (PMC6837288; doi:10.1371/journal.pgen.1008420)
Supplement: S1 Text — (DOCX) [file pgen.1008420.s001.docx]

Supplementary Information Text

**Supplemental Results**

*Population genetic analyses*

Using 296,196 bi-allelic sites genotyped in Mount Evans, Lincoln, and Merced mice, we assessed population structure using principal components analysis (PCA) and admixture. On a plot of the first two principal components (PC; **Supplemental Fig. 2**), the three populations form distinct clusters, with PC1 separating east to west (i.e. Lincoln from California with Mt Evans intermediary) and accounting for 9.03% total variation. PC2 separated Mt Evans from Lincoln and California populations and accounted for the next 7.61% of variation. Admixture analysis showed a similar pattern to PCA (**Supplemental Fig. 3**), with Lincoln separating from Merced and Mount Evans at K=2, and all three populations separating out at K=3. Cross validation showed the lowest error at K=1, yet K=2 and K=3 supported biologically significant populations. These results suggest that Merced and Lincoln are appropriate populations to use for calculating PBS values within Mount Evans.

*Cline analysis*

To assess the geographic and phylogenetic extent of the coding ^Thr^762^Met^ Epas1 SNP, we genotyped 229 deer mouse samples collected from 23 sampling sites across the western US, and found that higher elevation populations have a higher frequency of the ^Thr^762^Met^ allele (**Fig.** **2A**), and the frequency of the Epas1^H^ allele is significantly and positively correlated (r^2^=0.589, p<0.001) with elevation (**Supplemental Fig. 6**). For a single elevational transect connecting Lincoln to Mt. Evans, variation in Epas1 allele frequency was best explained as a sigmoidal cline with a cline centered at 1399.5 m a.s.l. (95% CI 1192.99 – 1493.01 m a.s.l.), with a width of 529.3 m (95% CI 144.65 – 881.07 m) (**Fig.** **2C**). Based on the phylogeny, the 762^Met^ (henceforth called the *Epas1^H^* allele) appears to be derived in Mount Evans mice with respect to ancestral Lincoln mice and their sister species *P. polionotus* (**Supplemental Table 2;** ^1^).

*Ventilatory and metabolic responses to acute hypoxia*

All *Epas1* genotypes exhibited similar ventilatory and metabolic responses to acute hypoxia challenge (**Supplemental Fig. 11**, **Supplemental Fig. 12**; **Supplemental Table 6**). Total ventilation increased as the level of hypoxia became more severe (main effect of PO_2_: F_4,148_=20.385, P<0.001), but it was not influenced by *Epas1* genotype (F_2,148_=0.3140, P=0.7325). This hypoxic ventilatory response was driven by a significant increase in breathing frequency with decreasing PO_2_ (F_4,148_=218.68, P<0.001), offset somewhat by more modest decreases in tidal volume (F_4,148_=42.669, P<0.001), and occurred in association with a significant drop in arterial O_2_ saturation (F_4,148_=306.47, P<0.001). However, *Epas1* genotype had no effect on breathing frequency (F_2,148_=0.0460, P=0.9548), tidal volume (F_2,148_=0.2680, P=0.7660), or SaO_2_ (F_2,148_=2.1270, P=0.1332). Hypoxia also depressed V̇O_2_ (F_4,148_=21.782, P<0.001) and T_b_ (F_1,74_=56.895, P<0.001), but neither variable was affected by genotype (F_2,148_=0.8770, P=0.4245; F_2,74_=1.3510, P=0.2715). V̇CO_2_ also decreased in hypoxia, but neither V̇CO_2_ and respiratory exchange ratio (V̇CO_2_/V̇O_2_) were affected by *Epas1* genotype (data not shown). Several other calculated respiratory variables were also unaffected by *Epas1* genotype (ventilatory equivalent for O_2_, F_2,148_=0.4590, P=0.6352; pulmonary O_2_ extraction, F_2,148_=0.5627, P=0.5744). Hypoxic, cold-induced V̇O_2_ max (thermogenic capacity) also did not differ between *Epas1* genotype in 2016 (ANCOVA; F_2,22_=0.9; P>0.05), and so was not measured in 2017. Average mass-corrected thermogenic capacity values (V̇O_2_ max / body mass) ± standard error of the mean are as follows: *Epas1^L/L^* (n=3): 0.28 ± 0.01; *Epas1^H/L^* (n=9): 0.28 ± 0.02; *Epas1^H/H^(n=20):* 0.27 ± 0.01.

In contrast to the above respiratory and metabolic variables, the heart rate response to hypoxia was altered by *Epas1* genotype (**Fig. 2, Supplemental Fig. 13**). Heart rate increased in moderate hypoxia (12 and 10 kPa O_2_) in *Epas1*^H/H^ mice, but not in *Epas1*^H/L^ or *Epas1*^L/L^ mice, and there was a significant main effect of genotype on heart rate (F_2,148_=3.8240, P=0.0309). There was a significant effect of *Epas1* genotype on the increase in heart rate from 21 to 12 kPa O_2_ (the PO_2_ at the summit of Mount Evans) (F_2,37_=4.294, P=0.021) (**Supplemental Fig. 13**) and there was a significant pairwise difference between *Epas1*^H/H^ mice and *Epas1*^H/L^ mice (**Fig. 2**). However, heart rate declined similarly between genotypes as the level of hypoxia became more severe (main effect of PO_2_: F_4,148_=19.741, P<0.001), likely as a consequence of the depression in VO_2_ in severe hypoxia (**Fig.** **2**).

*Gastrocnemius muscle capillarity and enzyme activity*

*Epas1* genotype did not affect the capillarity or the activity of oxidative enzymes in the gastrocnemius muscle (**Supplemental Fig. 8, Supplemental Fig. 9**, **Supplemental Fig. 10, Supplemental Table 5**). *Epas1* genotype did not account for any statistically significant variation in capillary surface density (F_2,29_=0.2741, P=0.7622), the number of capillaries per muscle fibre (F_2,29_=0.1009, P=0.9043), capillary density (F_2,29_=0.1483, P=0.8628), or the average transverse area of muscle fibres (F_2,29_=0.0513, P=0.9501). *Epas1* genotype also had no effect on the activities of cytochrome c oxidase (COX; F_2,29_=2.0110, P=0.1521) or citrate synthase (CS; F_2,29_=0.7222, P=0.4942) in the muscle. However, there was a nearly significant main effect of *Epas1* genotype on lactate dehydrogenase (LDH) activity in the muscle (F_2,27_=2.7801, P=0.0798). In consideration of the fact that we may have been underpowered to detect significant differences in the *Epas1*^L/L^ genotype (n=4), we did a post-hoc comparison between only *Epas1*^H/H^ and *Epas1*^H/L^ genotypes, and thus detected a statistically significant difference in LDH activity between genotypes (F_1,24_=5.3261, P=0.0299). Otherwise, all haematological measurements and organ masses were similar between *Epas1* genotypes (**Supplemental Table 3, Supplemental Table 4**).
