# Supplementary material for: Physiological and genomic evidence that selection on the transcription factor Epas1 has altered cardiovascular function in high-altitude deer mice
Source: PLoS Genet. 2019 Nov 7;15(11):e1008420. doi: 10.1371/journal.pgen.1008420 (PMC6837288; doi:10.1371/journal.pgen.1008420)
Supplement: S1 Fig — Distribution of mean depth of coverage for 100 exome samples used in this study. The final set of quality-filtered sites consists of 5,182,530 high-quality bi-allelic variants sequenced at approximately 18X coverage. Red dashed vertical line indicates the mean. (PDF) [file pgen.1008420.s015.pdf]

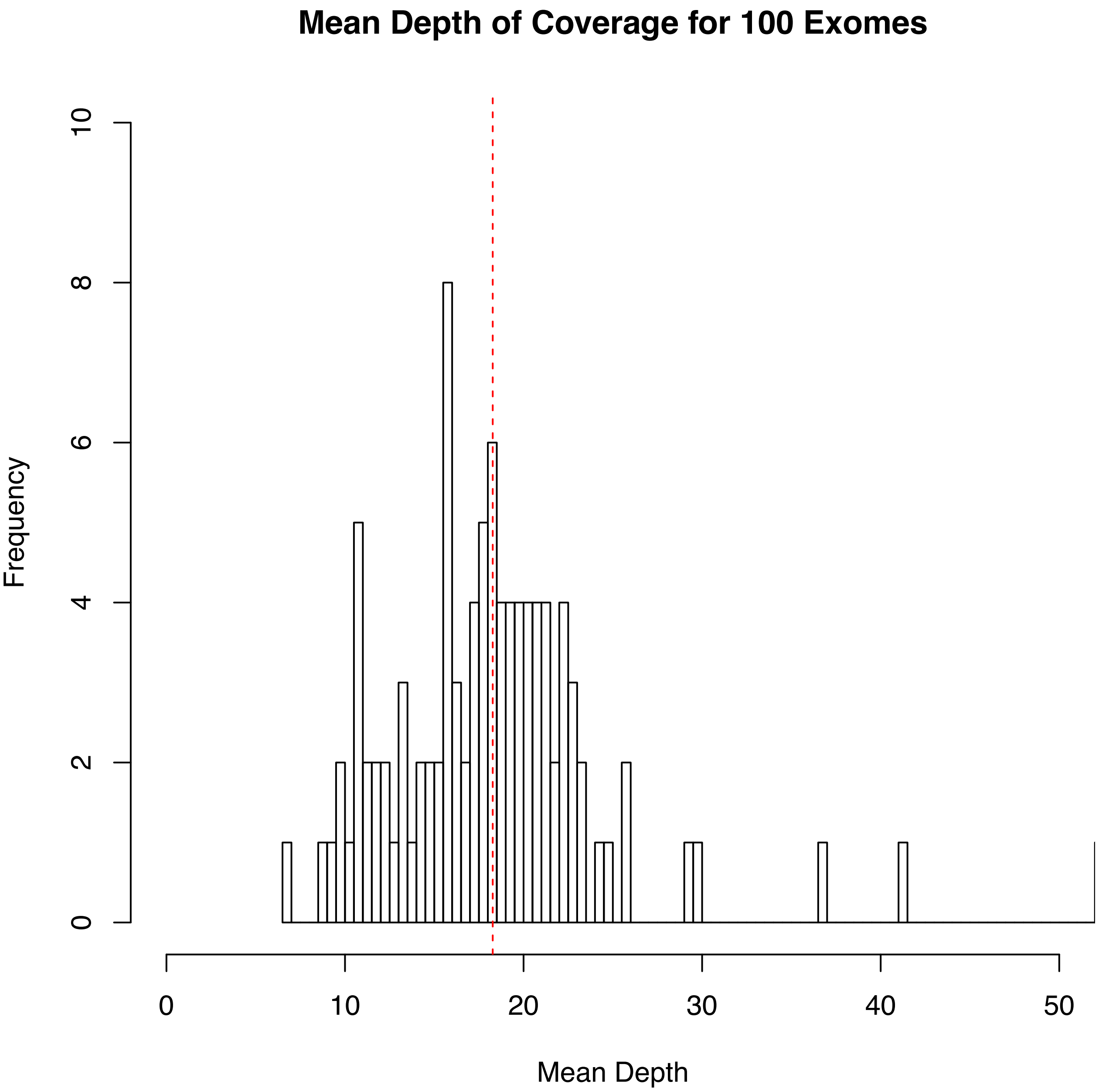

Figure S1. Distribution of mean depth of coverage for 100 exome samples used in this study. The final set of quality-filtered sites consists of 5,182,530 high-quality bi-allelic variants sequenced at approximately 18X coverage. Red dashed vertical line indicates the mean.

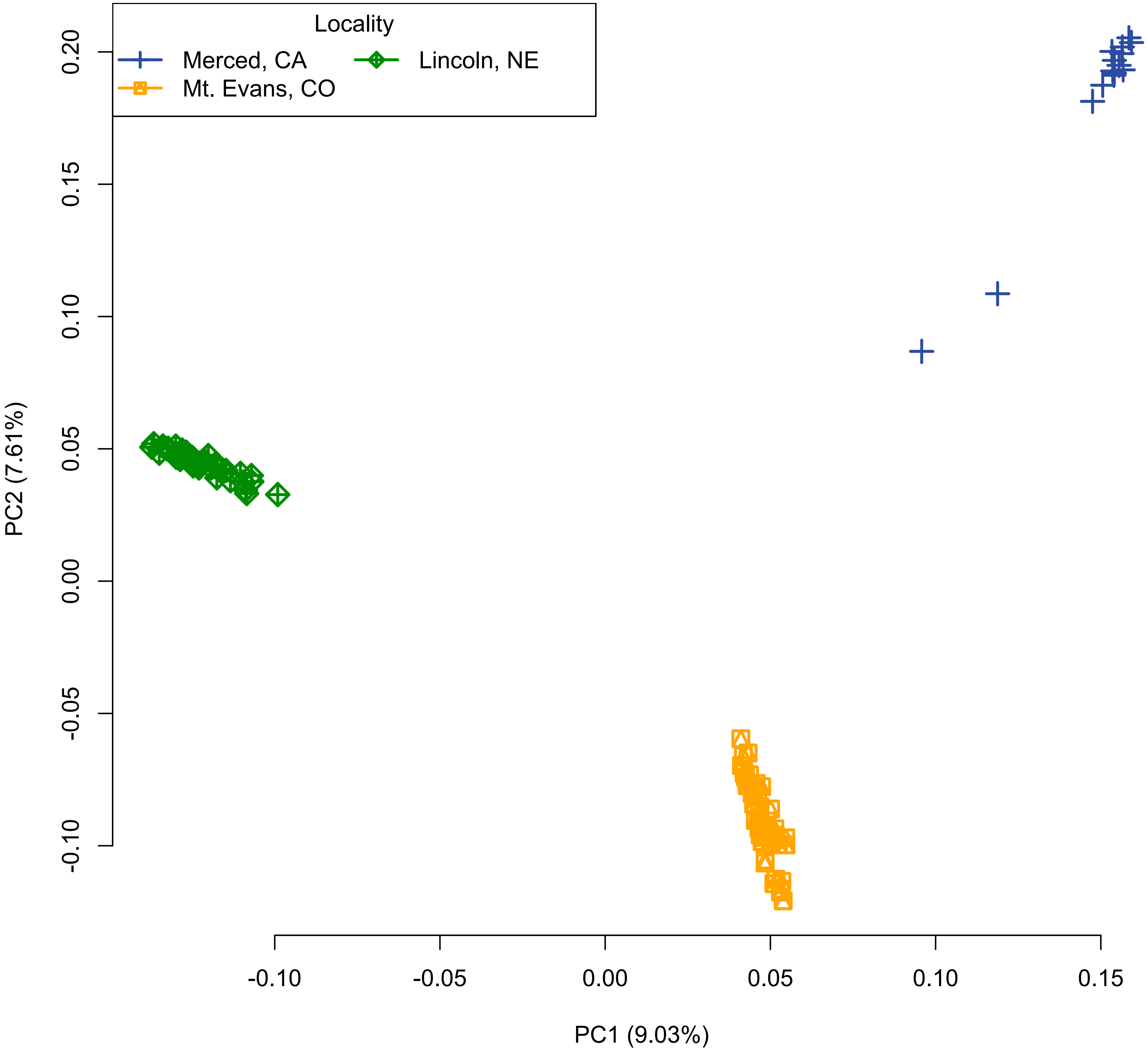

Figure S2. Principal components analysis of Mount Evans (n=48), Lincoln (n=37), and California (n=15) mice, based on genotypes from 296,196 exome-wide LD-pruned SNPs with no missing data.

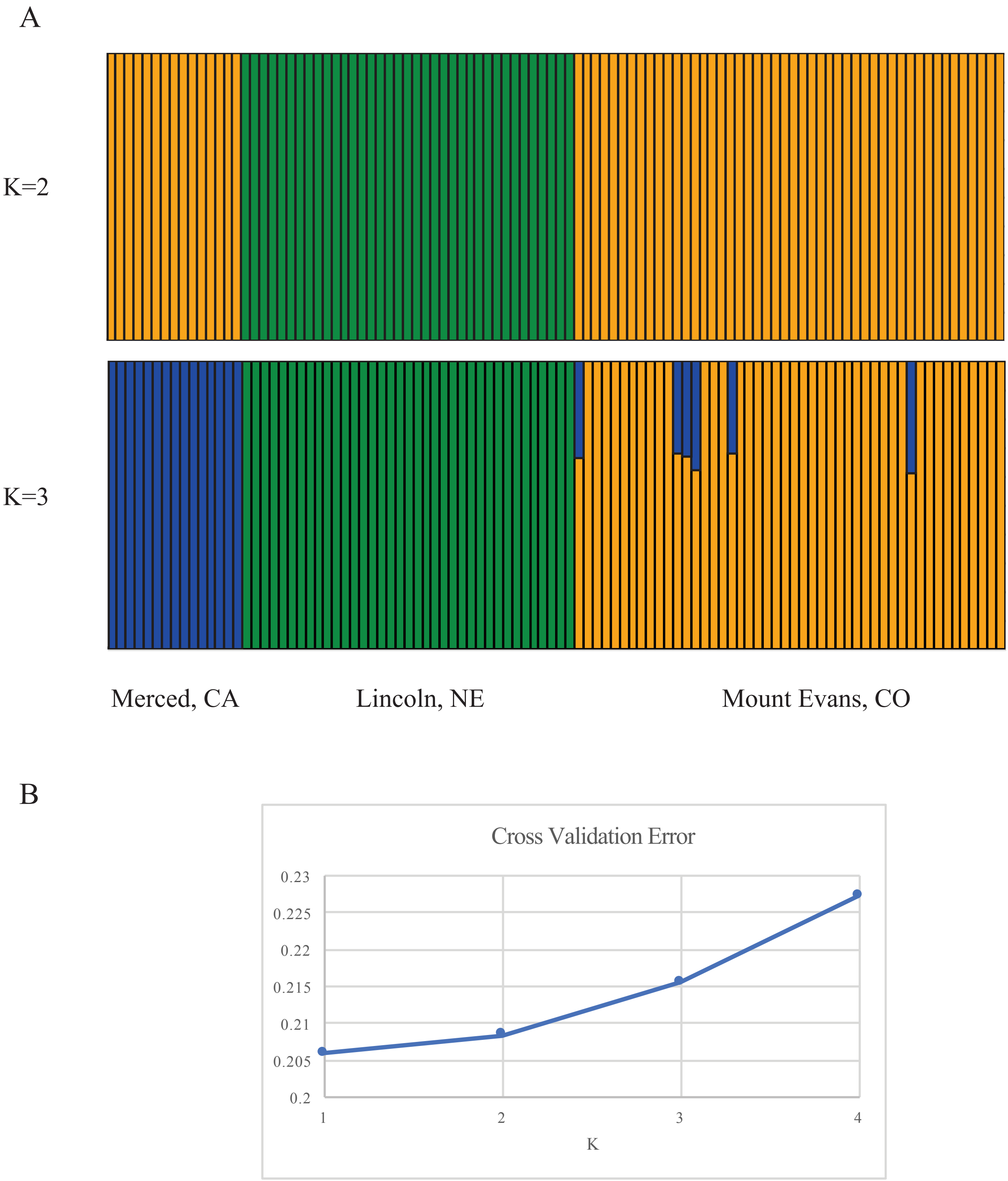

Figure S3. A) Population assignment made via Admixture for K=2 to K=3 for 100 individuals. Admixture was run on a set of 296,196 exome-wide LD-pruned SNPs with no missing data. B) The lowest cross validation error rate was at K=1; however, higher values of K are biologically meaningful and are therefore shown here.

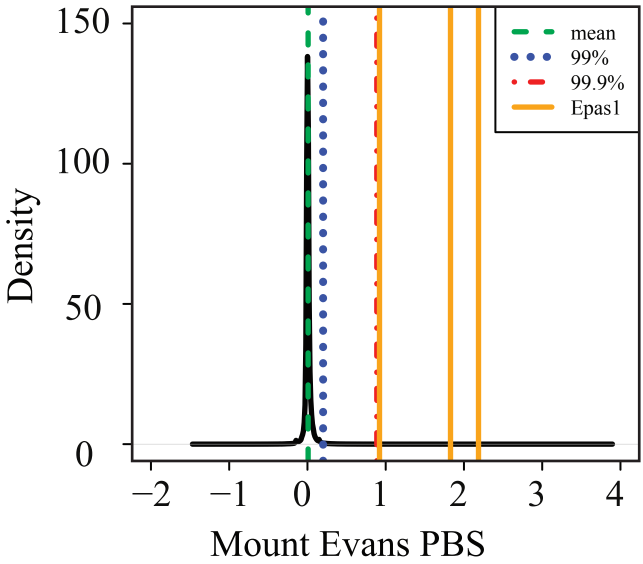

Figure S4. Density distribution of population branch statistic (PBS) values calculated for Mount Evans, using Lincoln and Merced populations as outgroups. The mean (green vertical dashed line), 99th (blue vertical dotted line), and 99.9th (red dash-dotted line) values of the empirical distribution are shown. Orange vertical lines indicate three outlier SNPs located in Epas1, with the rightmost line indicating the Thr762Met SNP.

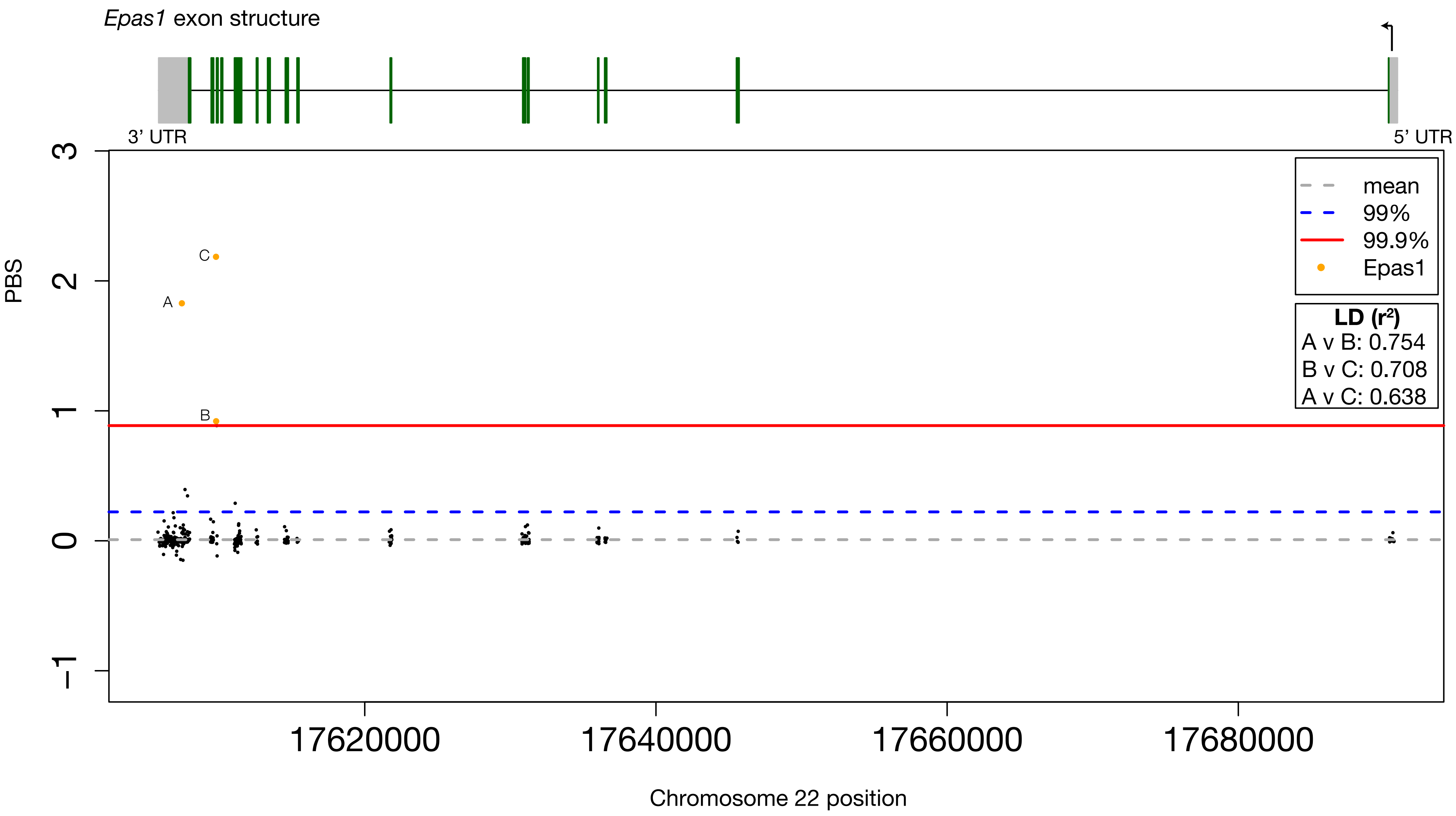

Figure S5. Manhattan plot of PBS values for all SNPs (black dots) located within all exons of *Epas1*. Exome-wide values for mean, 99%, and 99.9% percentile PBS values are shown, and three outlier SNPs above the 99.9th percentile located in Epas1 are highlighted in orange. Pairwise linkage disequilibrium estimates (measured with the squared correlation coefficient, r^2) for each SNP pair are provided.

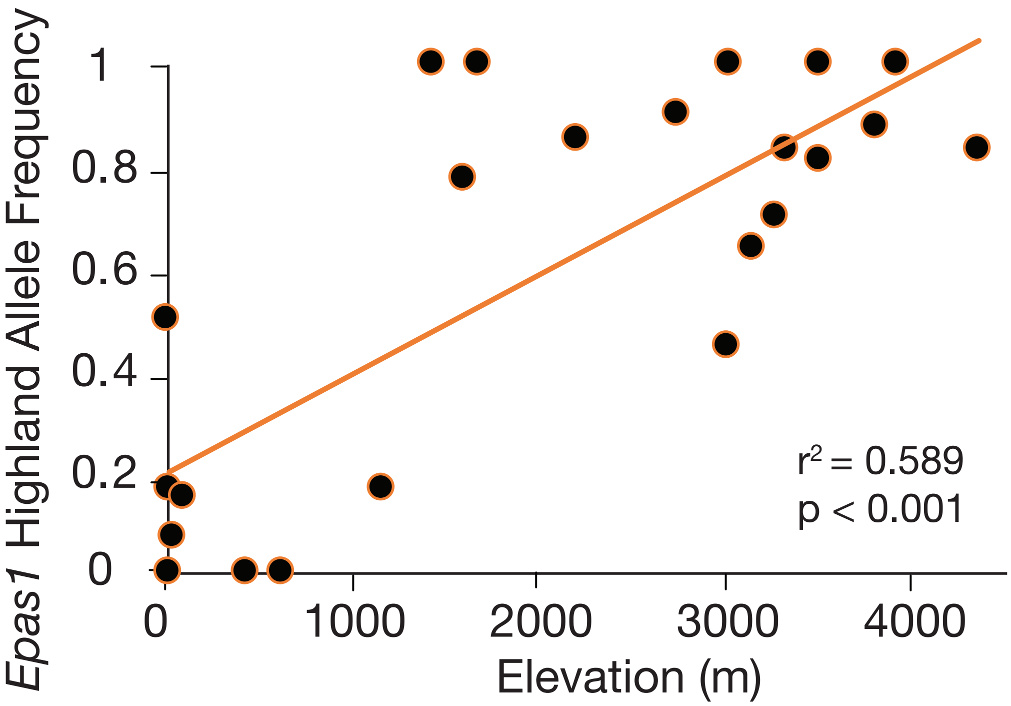

Figure S6. Significant positive correlation of high-elevation allele frequency with sampled elevation, based on genotyping 23 populations.

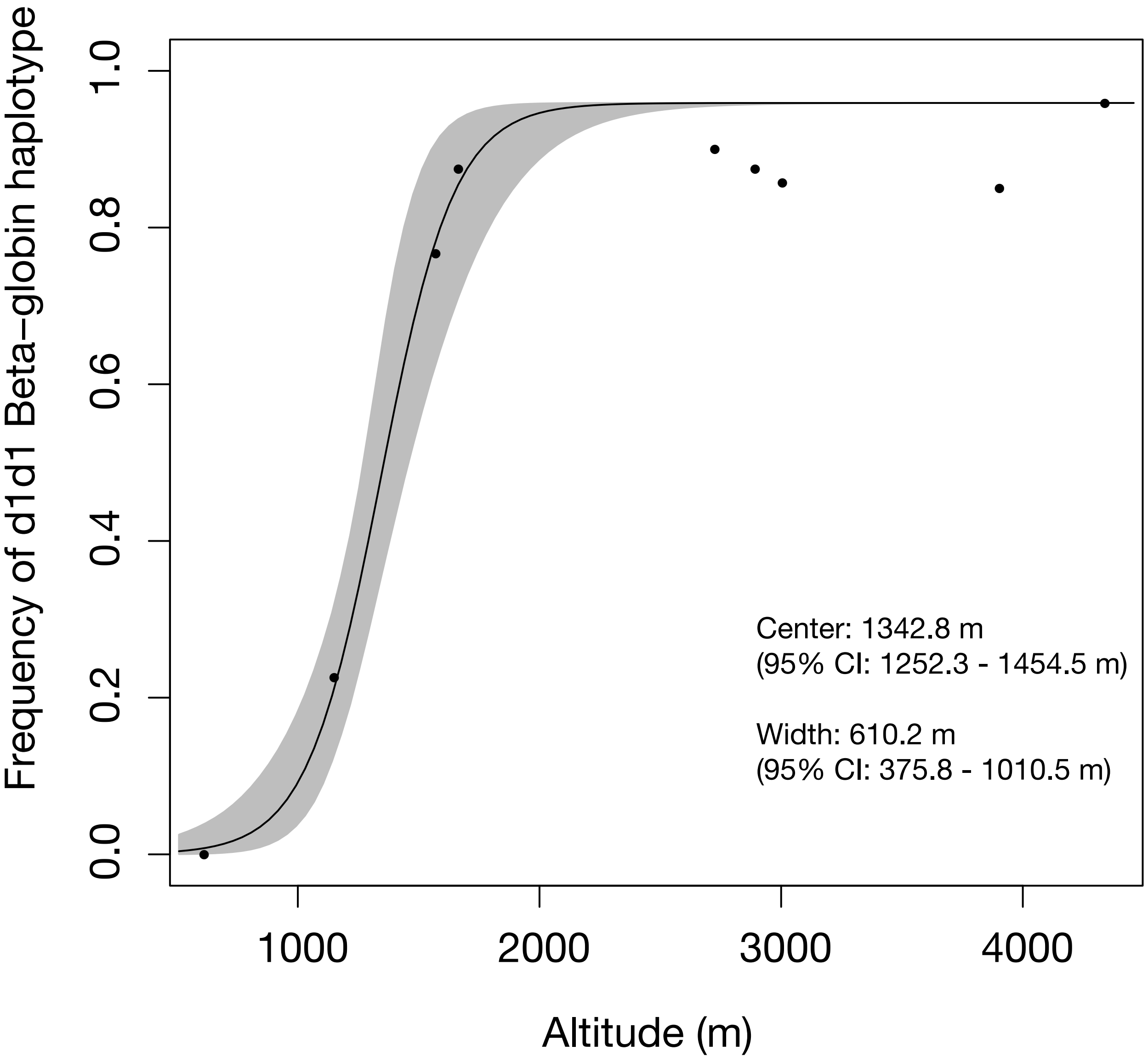

Figure S7. Clinal variation in two-locus HBB haplotype frequencies for nine *P. maniculatus* populations sampled along a 4500 m altitudinal cline from the Great Plains of Nebraska to the Rocky Mountains in Colorado. Data from Storz *et al.* 2012 Genetics.

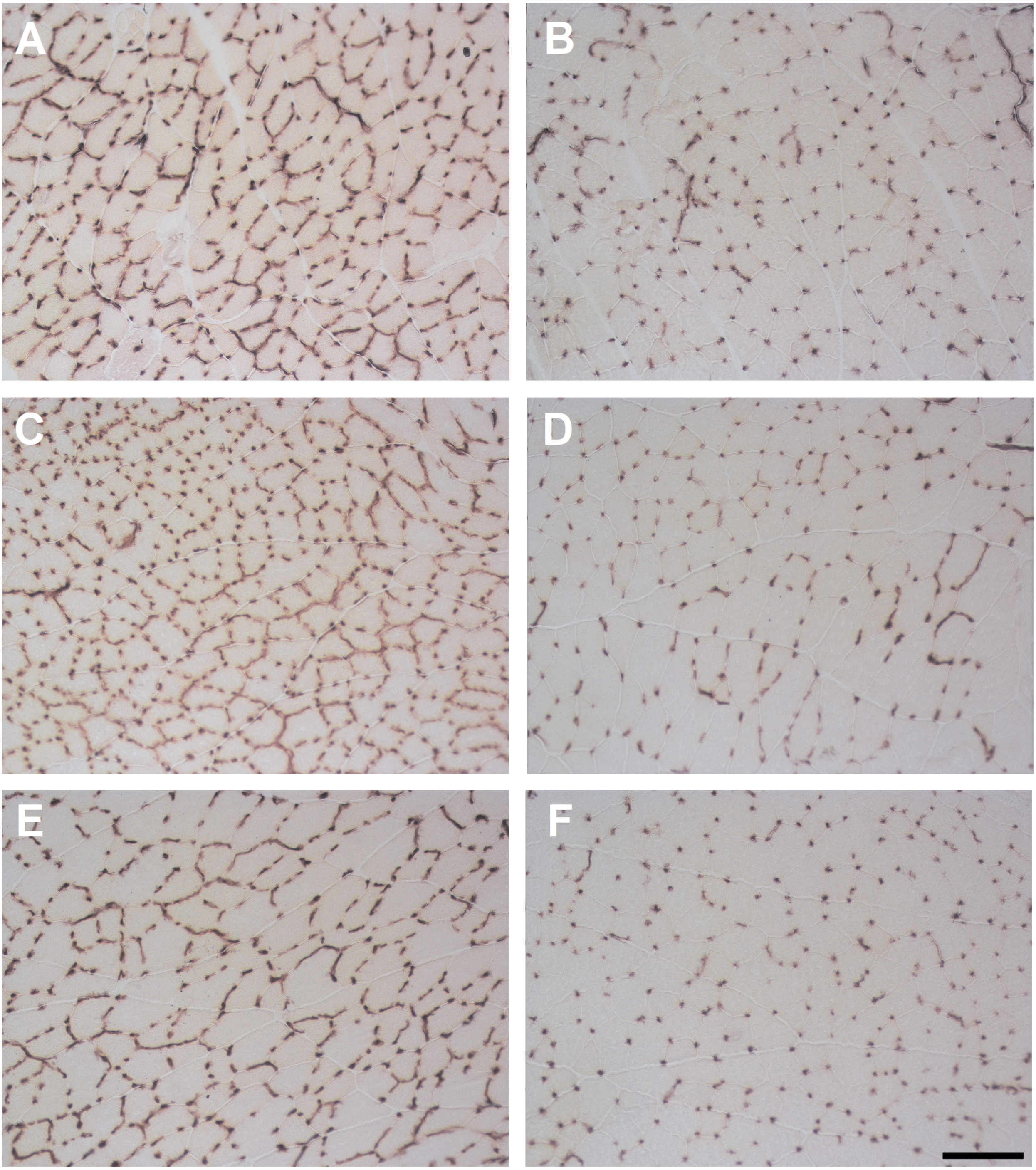

Figure S8. Histological analysis of capillarity in the gastrocnemius muscle. Capillaries were identified by staining for alkaline phosphatase activity. The oxidative core (A,C,E) and the outer less oxidative region (B,D,F) of the muscle is shown for representative individuals possessing *Epas1*$^{H/H}$ (A,B), *Epas1*$^{H/L}$ (C,D), and *Epas1*$^{L/L}$ (E,F) genotypes. All images are shown at the same scale, and the scale bar represents 100 μm.

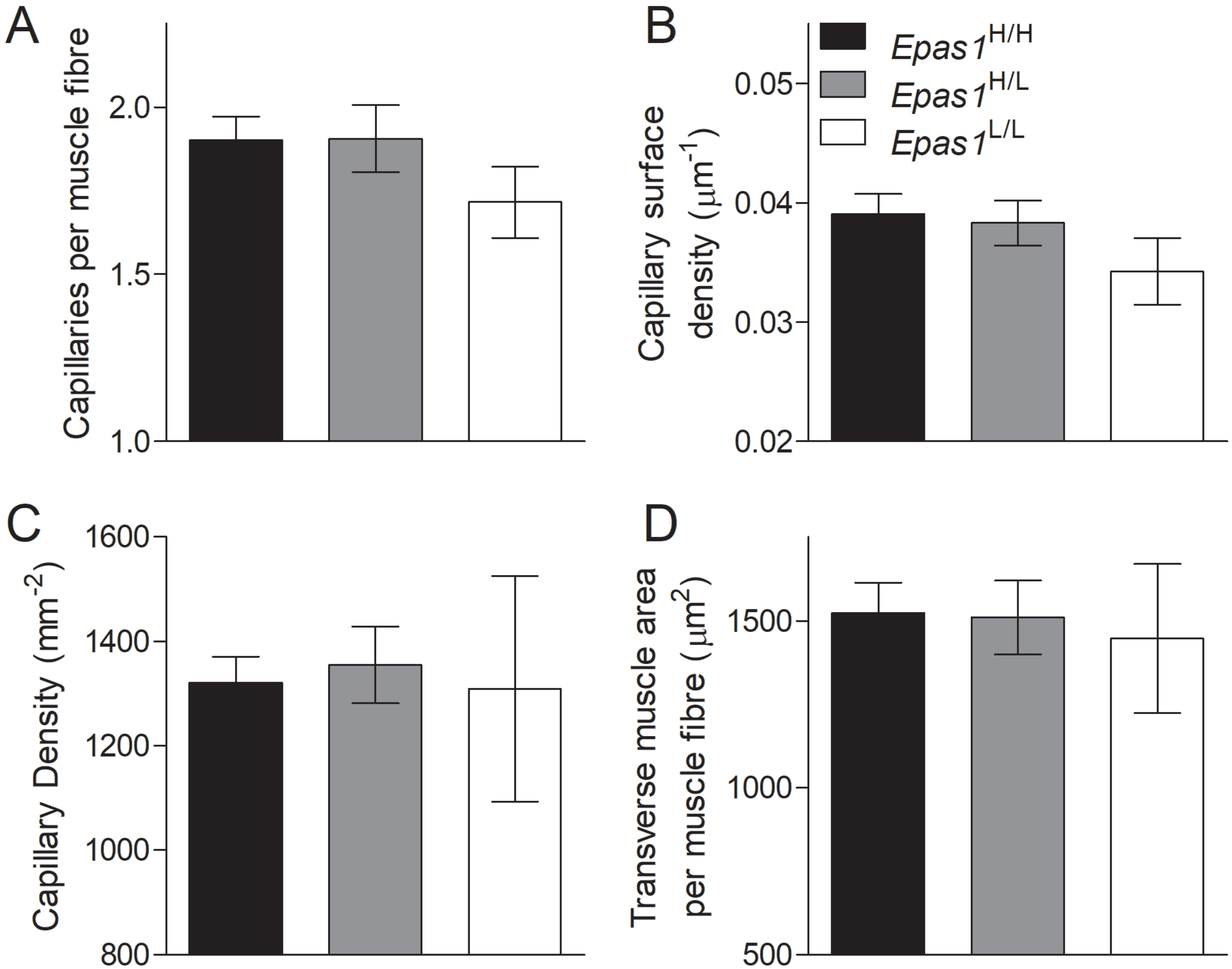

Figure S9. There were no differences in capillarity in the gastrocnemius muscle between between deer mice with different *Epas1* genotypes. n=16 *Epas1*$^{H/H}$, n=13 *Epas1*$^{H/L}$, and n=4 *Epas1*$^{L/L}$ variants.

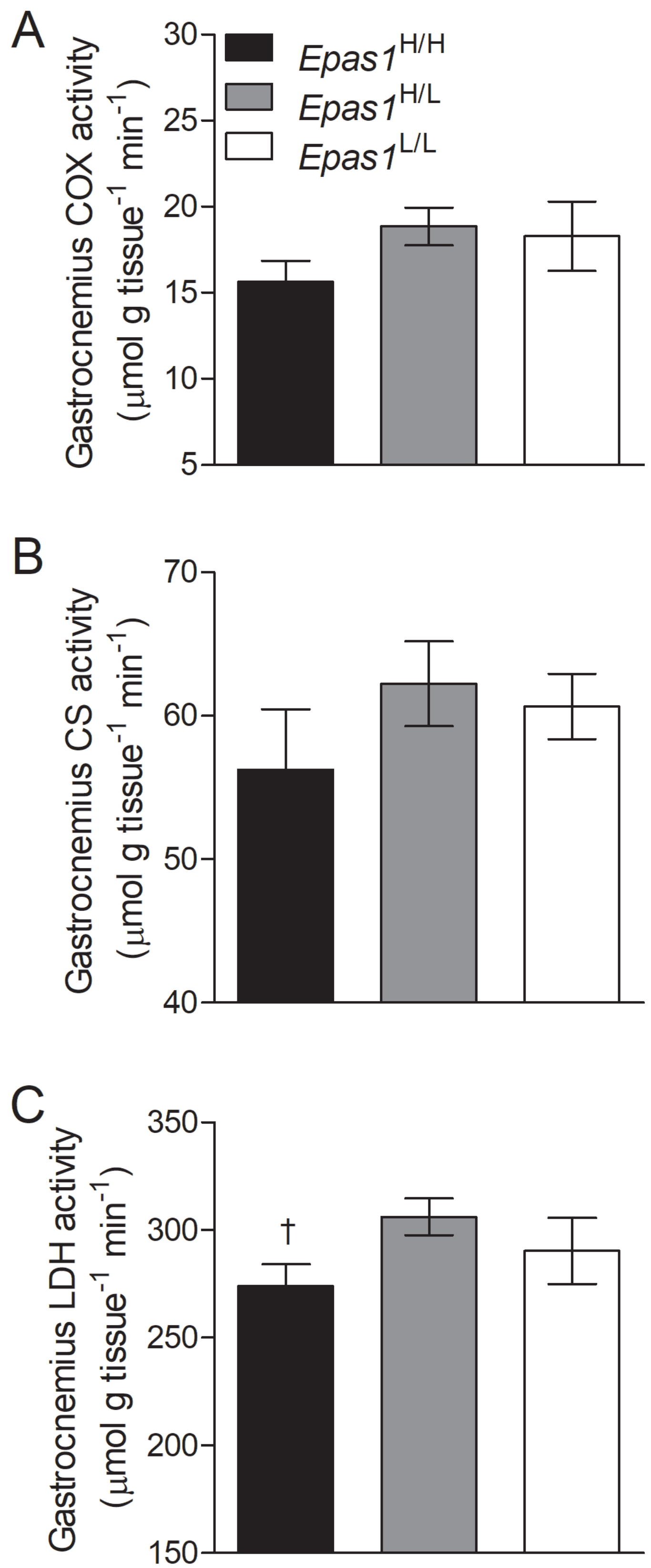

Figure S10. The activities of oxidative enzymes (cytochrome c oxidase, COX; citrate synthase, CS) in the gastrocnemius muscle were similar between deer mice with different *Epas1* genotypes, but lactate dehydrogenase (LDH) activity appeared to be lower in mice that were homozygous for the highland *Epas1* variant. † Significant difference in a post-hoc comparison between only $Epas1^{H/H}$ and $Epas1^{H/L}$ genotypes. n=16 $Epas1^{H/H}$, n=13 $Epas1^{H/L}$, and n=4 $Epas1^{L/L}$ variants.

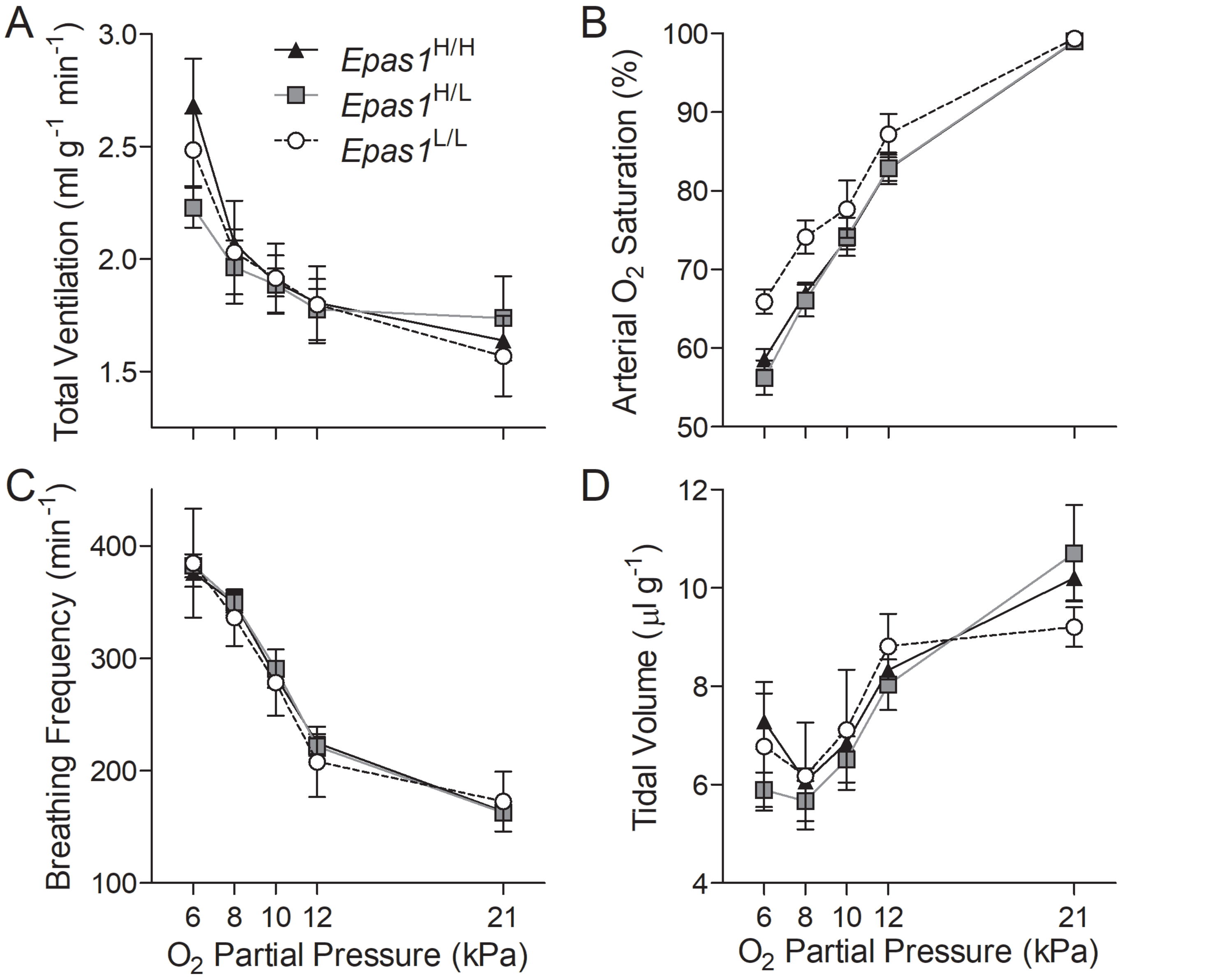

Figure S11. Deer mice with different *Epas1* genotypes exhibited similar ventilatory responses to increasingly severe levels of acute hypoxia. n=26 *Epas1*$^{H/H}$, n=13 *Epas1*$^{H/L}$, and n=4 *Epas1*$^{L/L}$ variants.

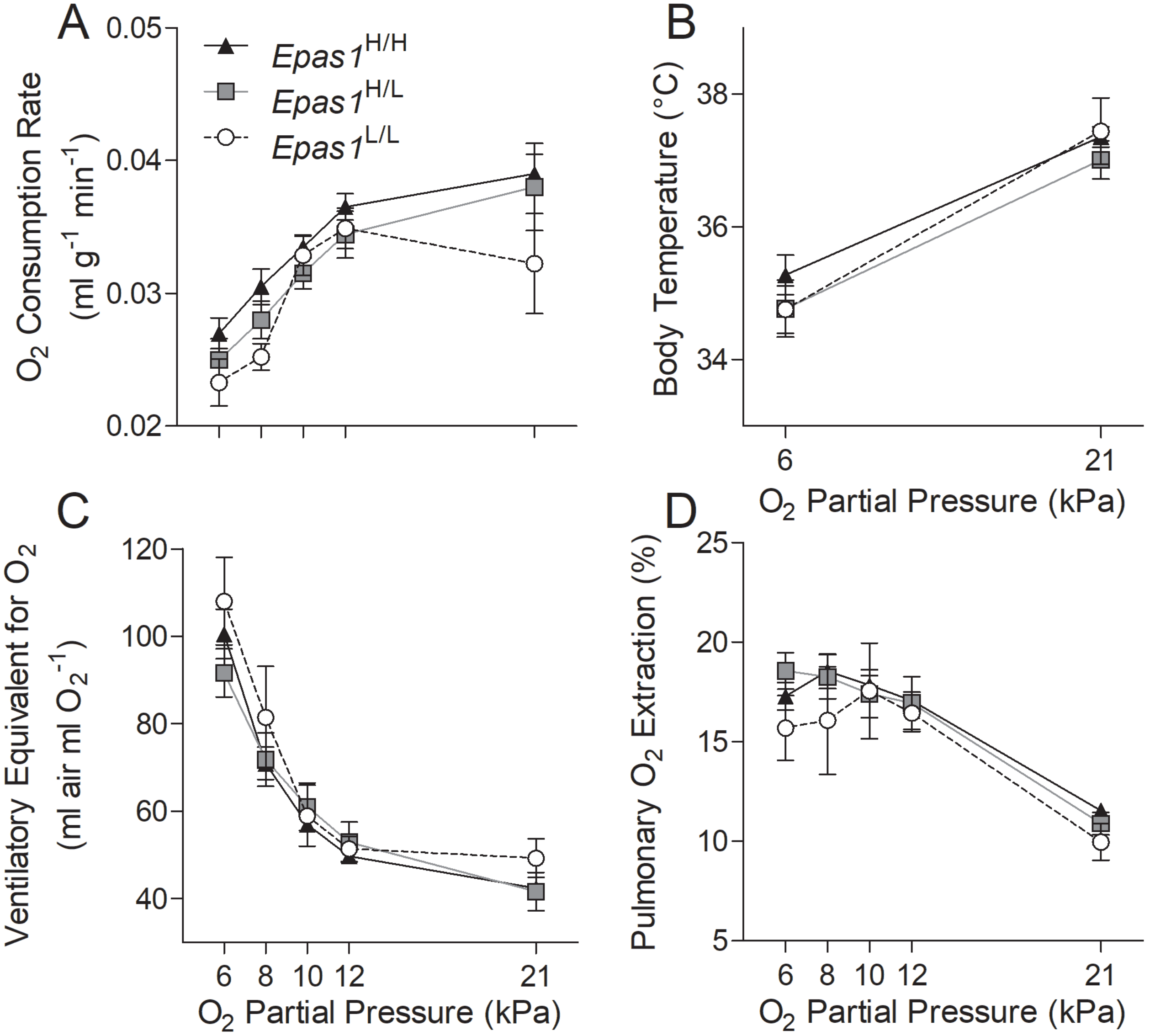

Figure S12. Deer mice with different *Epas1* genotypes exhibited similar declines in O_2 consumption rate and body temperature in response to increasingly severe levels of acute hypoxia, and similar increases in ventilatory equivalent for O_2 and pulmonary O_2 extraction. n=26 *Epas1*$^{H/H}$, n=13 *Epas1*$^{H/L}$, and n=4 *Epas1*$^{L/L}$ variants.

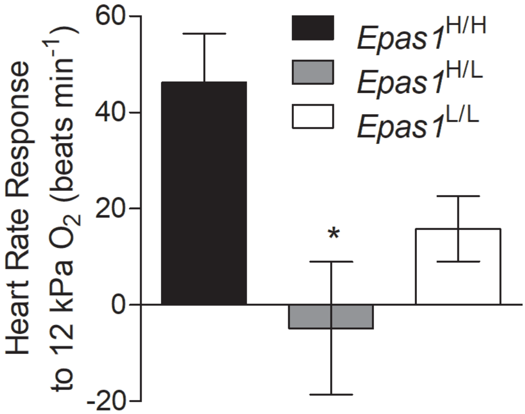

Figure S13. Deer mice that were homozygous for the highland *Epas1* variant exhibited a significantly greater increase in heart rate from normoxia (21 kPa O_2) to environmentally realistic levels of hypoxia at 4300 m elevation (12 kPa O_2). Measurements were made using a MouseOx Plus collar. * A significant pairwise difference between *Epas1*$^{H/H}$ and *Epas1*$^{H/L}$ mice. n=26 *Epas1*$^{H/H}$, n=13 *Epas1*$^{H/L}$, and n=4 *Epas1*$^{L/L}$ variants.

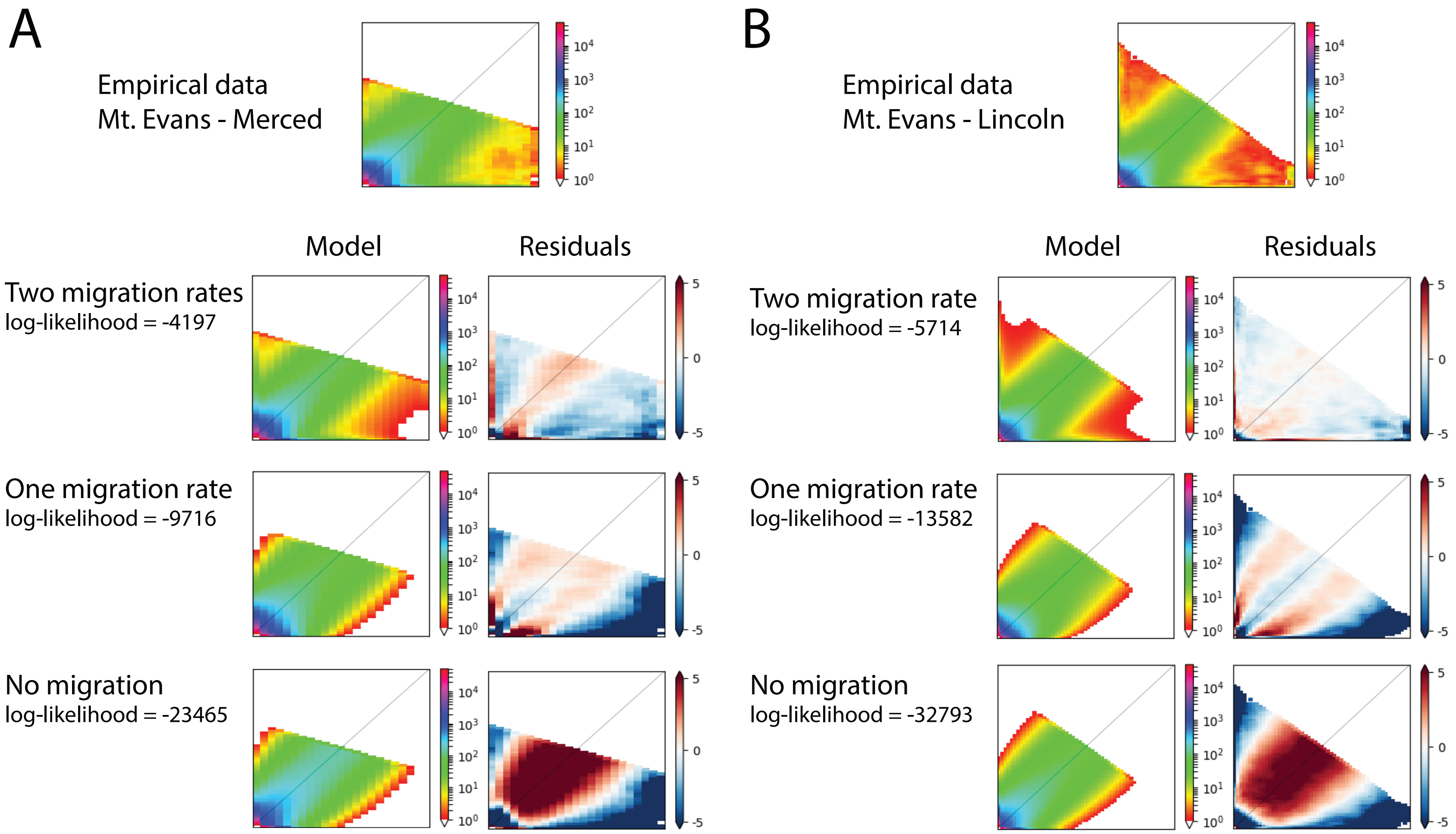

Figure S14. The folded 2-dimensional site frequency spectra (2d-SFS) for deer mice from (A) Mt. Evans, CO, and Merced, CA, and (B) Mt. Evans, CO, and Lincoln, NE. For each pair of populations, we show the empirical 2d-SFS from whole exome data and the maximum likelihood 2d-SFS for demographic models with no migration, one migration rate, and two migration rates. Residuals reflect the overall fit of the model to the empirical data, where red indicates an overestimation of the number of SNPs by the model and blue reflects an underestimation.

A

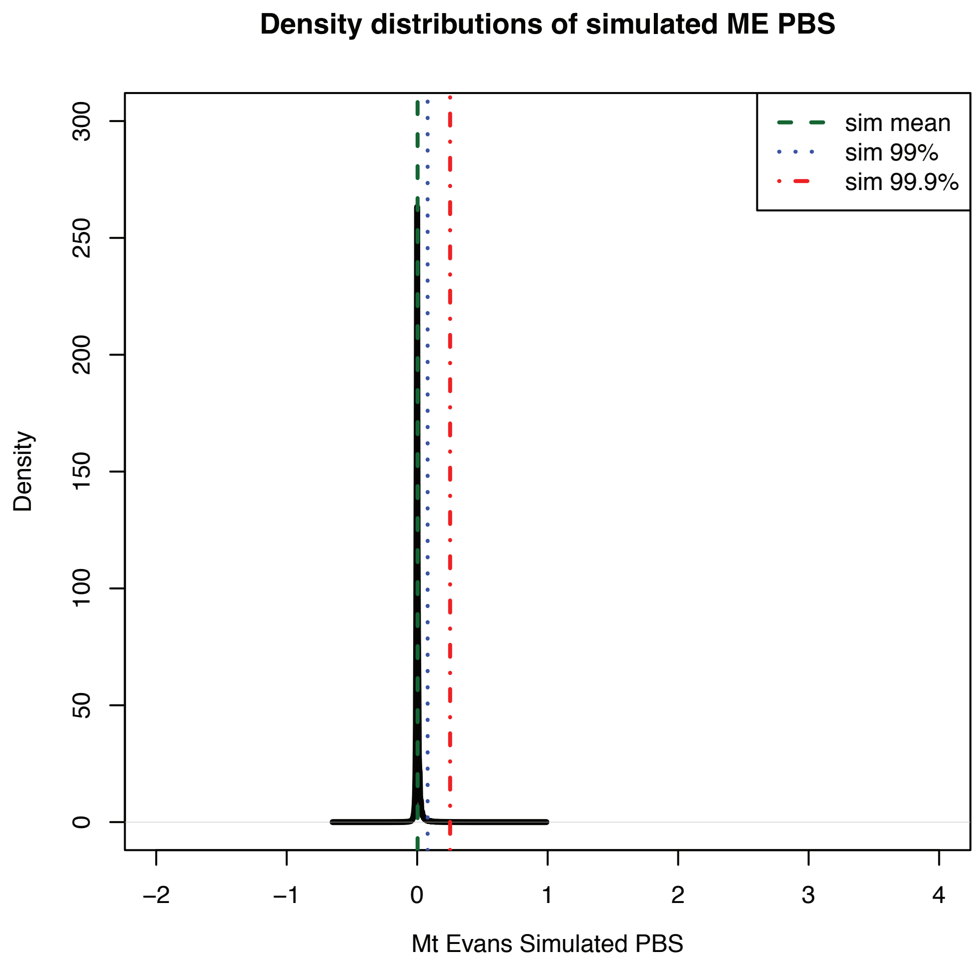

B

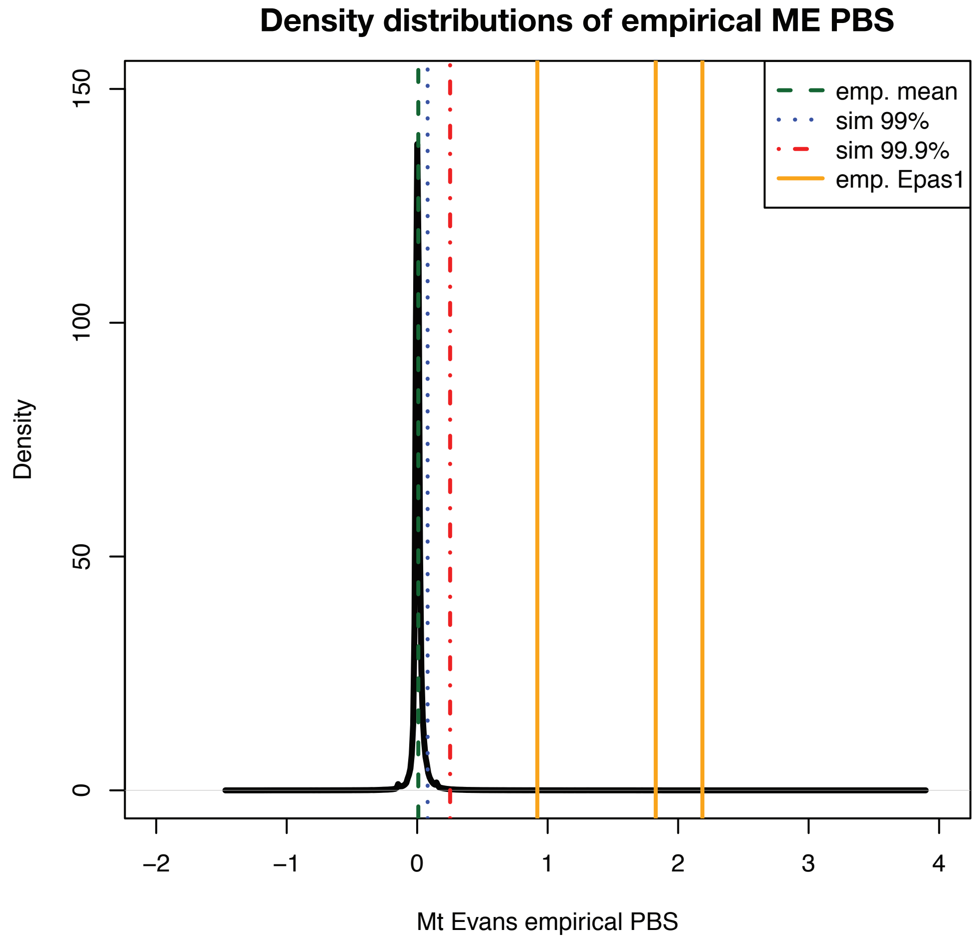

Figure S15. A) Density plot of population branch statistic (PBS) values calculated for Mount Evans, using Lincoln and Merced populations as outgroups, from 10,000 SNPs simulated under modeled demography. Values for the mean (green vertical dashed line), 99th (blue vertical dotted line), and 99.9th (red dash-dotted line) percentiles of the simulated distribution are shown. B) Density plot of empirical PBS values (same as in Figure S4), but with significance thresholds based on simulated 99th and 99.9th percentile shown.
