# Supplementary material for: Physiological and genomic evidence that selection on the transcription factor Epas1 has altered cardiovascular function in high-altitude deer mice
Source: PLoS Genet. 2019 Nov 7;15(11):e1008420. doi: 10.1371/journal.pgen.1008420 (PMC6837288; doi:10.1371/journal.pgen.1008420)
Supplement: S3 Fig — A) Admixture was run on a set of 296,196 exome-wide LD-pruned SNPs with no missing data. Each vertical bar represents an individual, with the colors corresponding to proportion assignment at each value of K. B) The lowest cross validation error rate was at K = 1; however, higher values of K are biologically meaningful and are therefore shown here. (PDF) [file pgen.1008420.s017.pdf]

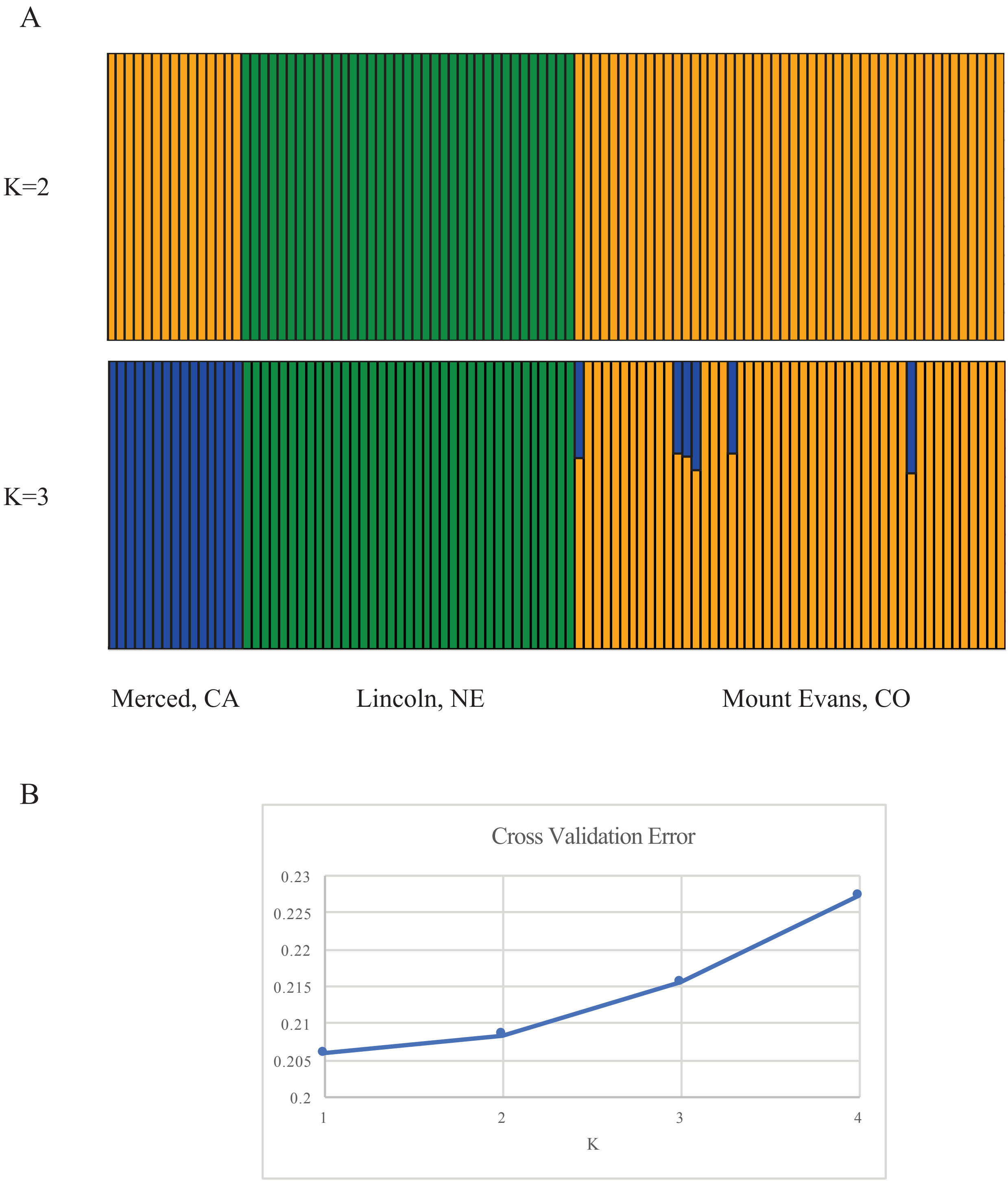

Figure S3. Population assignment made via Admixture for K=2 to K=3 for 100 individuals. A) Admixture was run on a set of 296,196 exome-wide LD-pruned SNPs with no missing data. Each vertical bar represents an individual, with the colors corresponding to proportion assignment at each value of K. B) The lowest cross validation error rate was at K=1; however, higher values of K are biologically meaningful and are therefore shown here.
