# Supplementary material for: Physiological and genomic evidence that selection on the transcription factor Epas1 has altered cardiovascular function in high-altitude deer mice
Source: PLoS Genet. 2019 Nov 7;15(11):e1008420. doi: 10.1371/journal.pgen.1008420 (PMC6837288; doi:10.1371/journal.pgen.1008420)
Supplement: S4 Fig — The mean (green vertical dashed line), 99th (blue vertical dotted line), and 99.9th (red dash-dotted line) values of the empirical distribution are shown. Orange vertical lines indicate three outlier SNPs located in Epas1, with the rightmost line indicating the Thr755Met SNP. (PDF) [file pgen.1008420.s018.pdf]

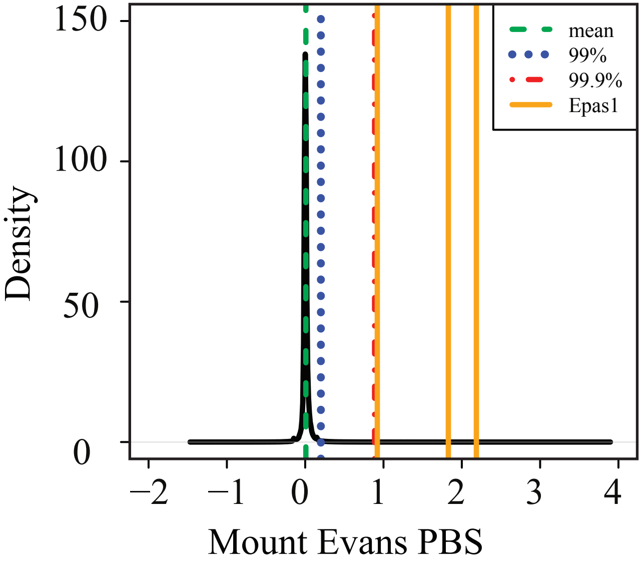

Figure S4. Density distribution of population branch statistic (PBS) values calculated for Mount Evans, using Lincoln and Merced populations as outgroups. The mean (green vertical dashed line), 99th (blue vertical dotted line), and 99.9th (red dash-dotted line) values of the empirical distribution are shown. Orange vertical lines indicate three outlier SNPs located in Epas1, with the rightmost line indicating the Thr762Met SNP.
