# Supplementary material for: Physiological and genomic evidence that selection on the transcription factor Epas1 has altered cardiovascular function in high-altitude deer mice
Source: PLoS Genet. 2019 Nov 7;15(11):e1008420. doi: 10.1371/journal.pgen.1008420 (PMC6837288; doi:10.1371/journal.pgen.1008420)
Supplement: S5 Fig — Manhattan plot of PBS values for all SNPs (black dots) located within all exons of Epas1. Exome-wide values for mean, 99%, and 99.9% percentile PBS values are shown, and three outlier SNPs above the 99.9th percentile located in Epas1 are highlighted in orange. Pairwise linkage disequilibrium estimates (measured with the squared correlation coefficient, r2) for each SNP pair are provided. (PDF) [file pgen.1008420.s019.pdf]

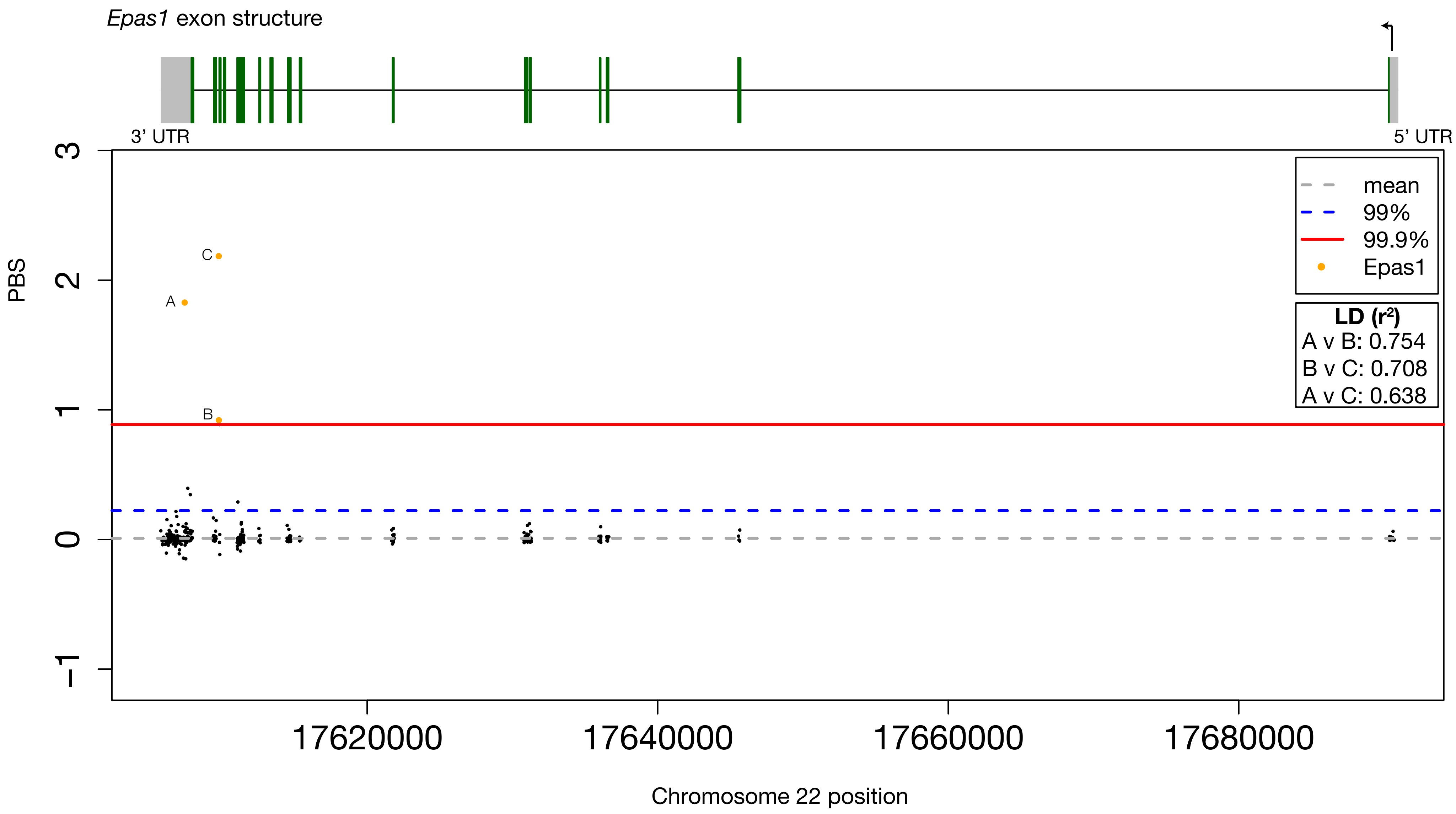

Figure S5. Manhattan plot of PBS values for all SNPs (black dots) located within all exons of *Epas1*. Exome-wide values for mean, 99%, and 99.9% percentile PBS values are shown, and three outlier SNPs above the 99.9th percentile located in Epas1 are highlighted in orange. Pairwise linkage disequilibrium estimates (measured with the squared correlation coefficient, r^2) for each SNP pair are provided.
