# Supplementary material for: Physiological and genomic evidence that selection on the transcription factor Epas1 has altered cardiovascular function in high-altitude deer mice
Source: PLoS Genet. 2019 Nov 7;15(11):e1008420. doi: 10.1371/journal.pgen.1008420 (PMC6837288; doi:10.1371/journal.pgen.1008420)
Supplement: S6 Fig — Significant positive correlation of high-elevation allele frequency with sampled elevation, based on genotyping 23 populations. (PDF) [file pgen.1008420.s020.pdf]

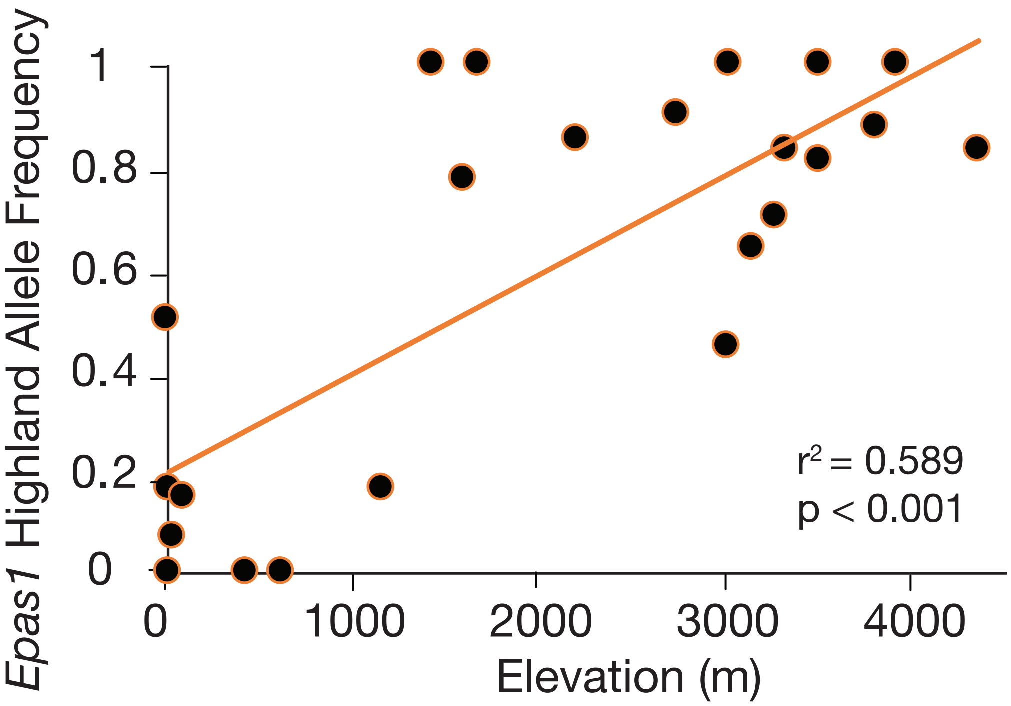

Figure S6. Significant positive correlation of high-elevation allele frequency with sampled elevation, based on genotyping 23 populations.
