# Supplementary material for: Physiological and genomic evidence that selection on the transcription factor Epas1 has altered cardiovascular function in high-altitude deer mice
Source: PLoS Genet. 2019 Nov 7;15(11):e1008420. doi: 10.1371/journal.pgen.1008420 (PMC6837288; doi:10.1371/journal.pgen.1008420)
Supplement: S7 Fig — Clinal variation for nine P. maniculatus populations sampled along a 4500 m altitudinal cline from the Great Plains of Nebraska to the Rocky Mountains in Colorado. Data from Storz et al. 2012 Genetics. (PDF) [file pgen.1008420.s021.pdf]

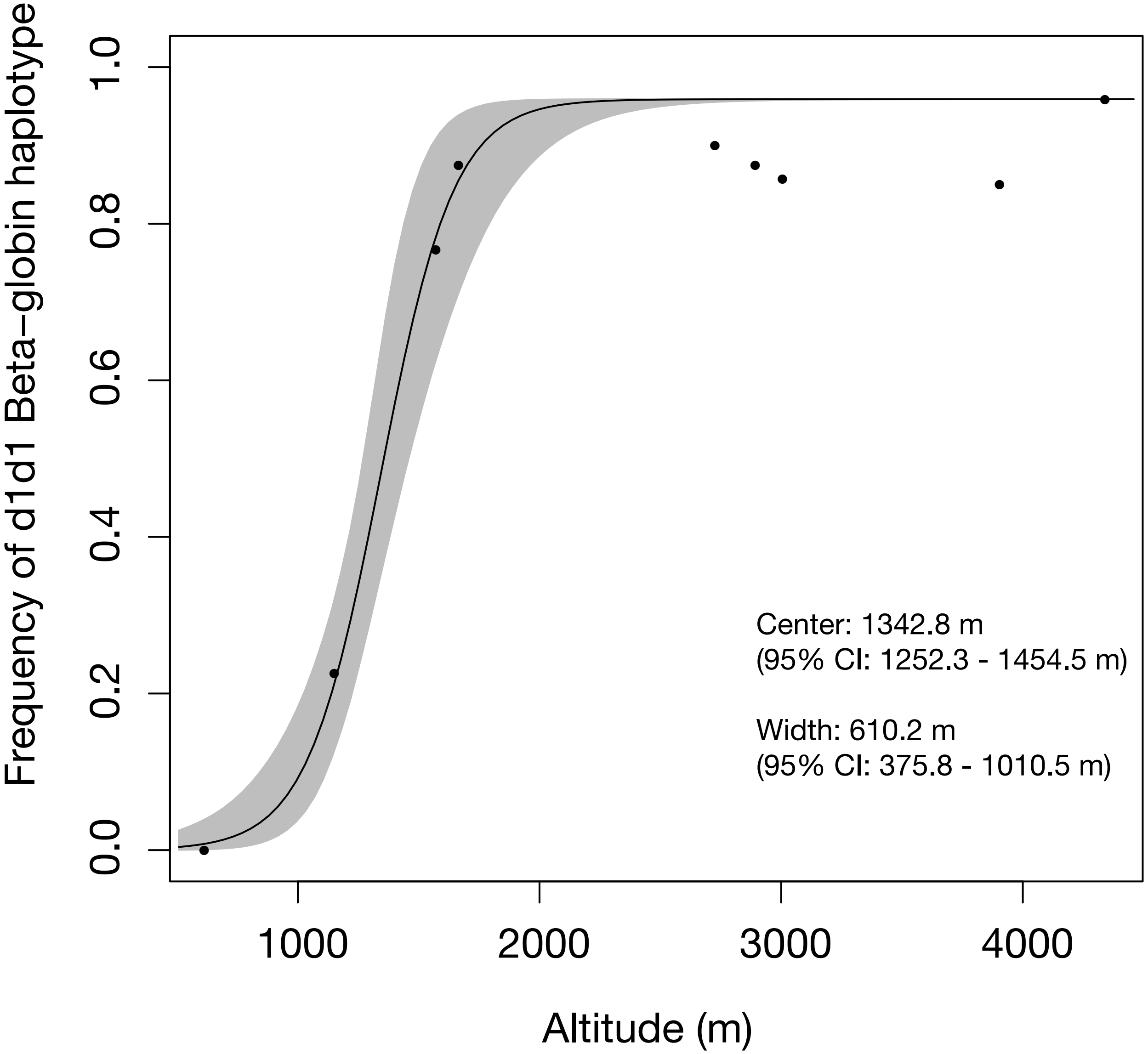

Figure S7. Clinal variation in two-locus HBB haplotype frequencies for nine *P. maniculatus* populations sampled along a 4500 m altitudinal cline from the Great Plains of Nebraska to the Rocky Mountains in Colorado. Data from Storz *et al.* 2012 Genetics.
