# Supplementary material for: Physiological and genomic evidence that selection on the transcription factor Epas1 has altered cardiovascular function in high-altitude deer mice
Source: PLoS Genet. 2019 Nov 7;15(11):e1008420. doi: 10.1371/journal.pgen.1008420 (PMC6837288; doi:10.1371/journal.pgen.1008420)
Supplement: S8 Fig — Capillaries were identified by staining for alkaline phosphatase activity. The oxidative core (A,C,E) and the outer less oxidative region (B,D,F) of the muscle is shown for representative individuals possessing Epas1H/H (A,B), Epas1H/L (C,D), and Epas1L/L (E,F) genotypes. All images are shown at the same scale, and the scale bar represents 100 μm. (PDF) [file pgen.1008420.s022.pdf]

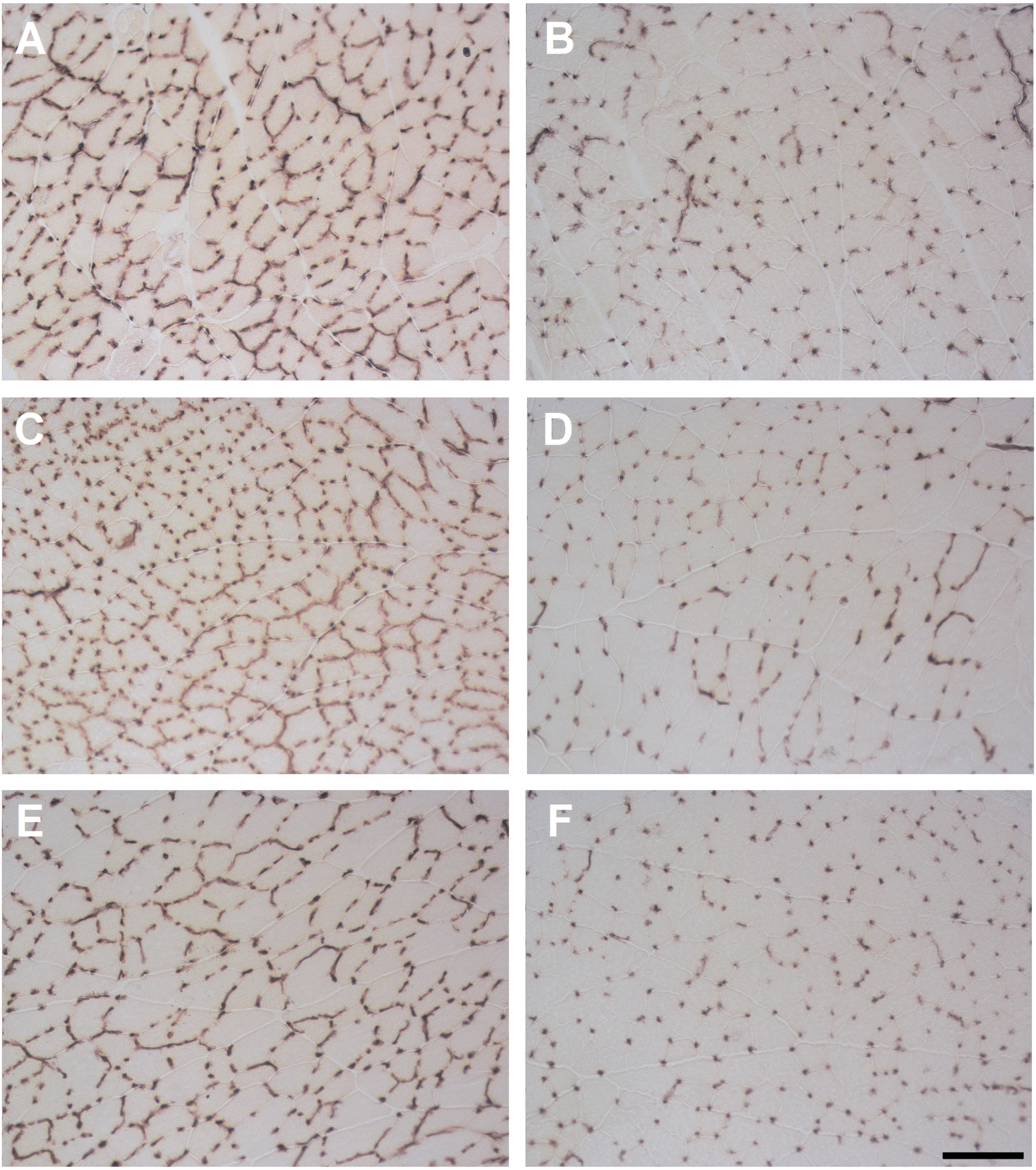

Figure S8. Histological analysis of capillarity in the gastrocnemius muscle. Capillaries were identified by staining for alkaline phosphatase activity. The oxidative core (A,C,E) and the outer less oxidative region (B,D,F) of the muscle is shown for representative individuals possessing *Epas1*$^{H/H}$ (A,B), *Epas1*$^{H/L}$ (C,D), and *Epas1*$^{L/L}$ (E,F) genotypes. All images are shown at the same scale, and the scale bar represents 100 µm.
