# Supplementary material for: Physiological and genomic evidence that selection on the transcription factor Epas1 has altered cardiovascular function in high-altitude deer mice
Source: PLoS Genet. 2019 Nov 7;15(11):e1008420. doi: 10.1371/journal.pgen.1008420 (PMC6837288; doi:10.1371/journal.pgen.1008420)
Supplement: S9 Fig — There were no differences in capillarity in the gastrocnemius muscle between deer mice with different Epas1 genotypes. Capillarity was quantified using the following measurements: A) capillaries per muscle fiber, B) capillary surface density, C) capillary density, and D) transverse muscle area per muscle fiber. Sample sizes: n = 16 Epas1H/H, n = 13 Epas1H/L, and n = 4 Epas1L/L variants. (PDF) [file pgen.1008420.s023.pdf]

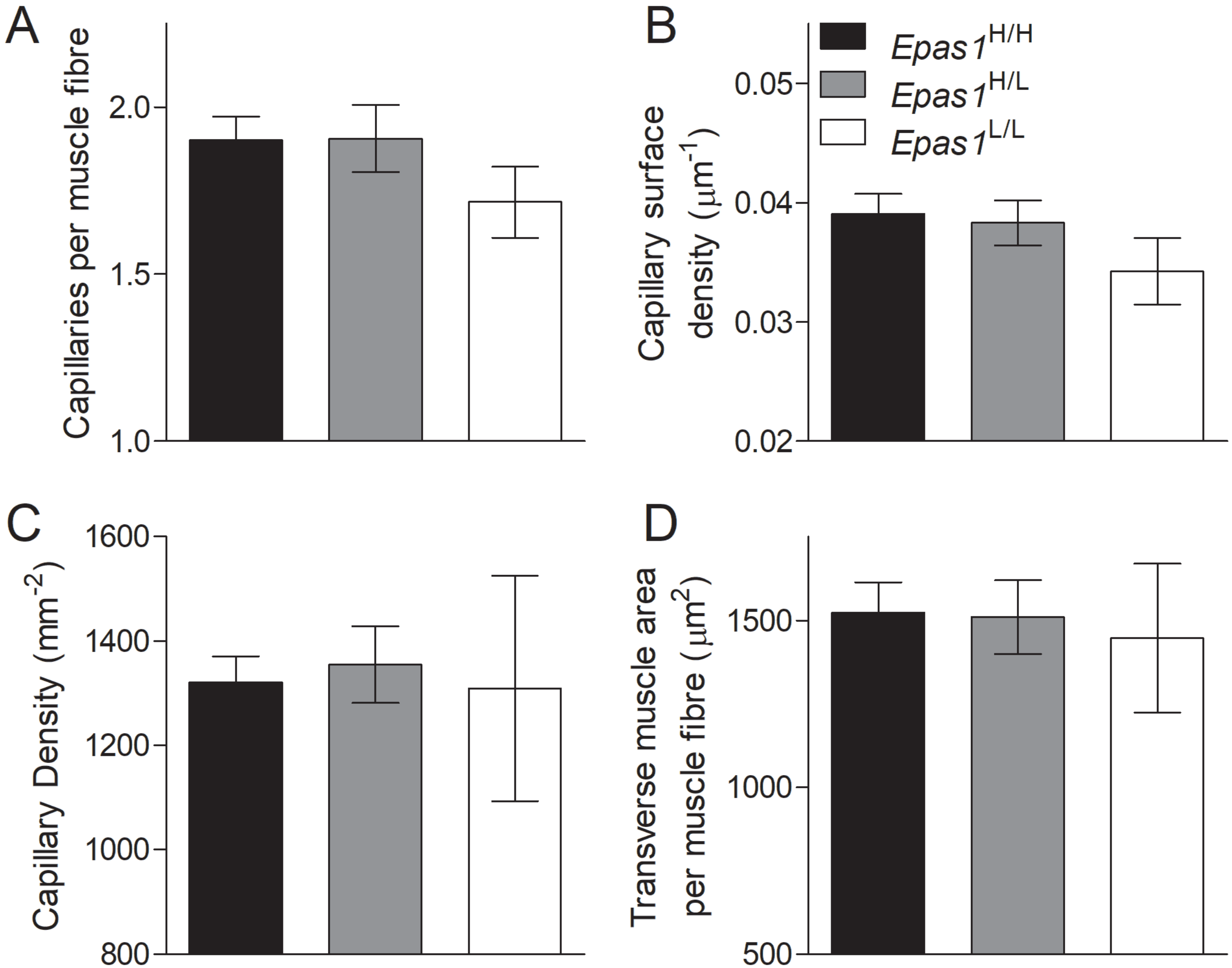

Figure S9. There were no differences in capillarity in the gastrocnemius muscle between deer mice with different *Epas1* genotypes. Capillarity was quantified using the following measurements: A) capillaries per muscle fiber, B) capillary surface density, C) capillary density, and D) transverse muscle area per muscle fiber. Sample sizes: n=16 *Epas1*$^{H/H}$, n=13 *Epas1*$^{H/L}$, and n=4 *Epas1*$^{L/L}$ variants.
