# Supplementary material for: Physiological and genomic evidence that selection on the transcription factor Epas1 has altered cardiovascular function in high-altitude deer mice
Source: PLoS Genet. 2019 Nov 7;15(11):e1008420. doi: 10.1371/journal.pgen.1008420 (PMC6837288; doi:10.1371/journal.pgen.1008420)
Supplement: S10 Fig — The activities of oxidative enzymes, i.e. A) cytochrome c oxidase (COX) and B) citrate synthase (CS) in the gastrocnemius muscle were similar between deer mice with different Epas1 genotypes, but C) lactate dehydrogenase (LDH) activity appeared to be lower in mice that were homozygous for the highland Epas1 variant. † Significant difference in a post-hoc comparison between only Epas1H/H and Epas1H/L genotypes. n = 16 Epas1H/H, n = 13 Epas1H/L, and n = 4 Epas1L/L variants. (PDF) [file pgen.1008420.s024.pdf]

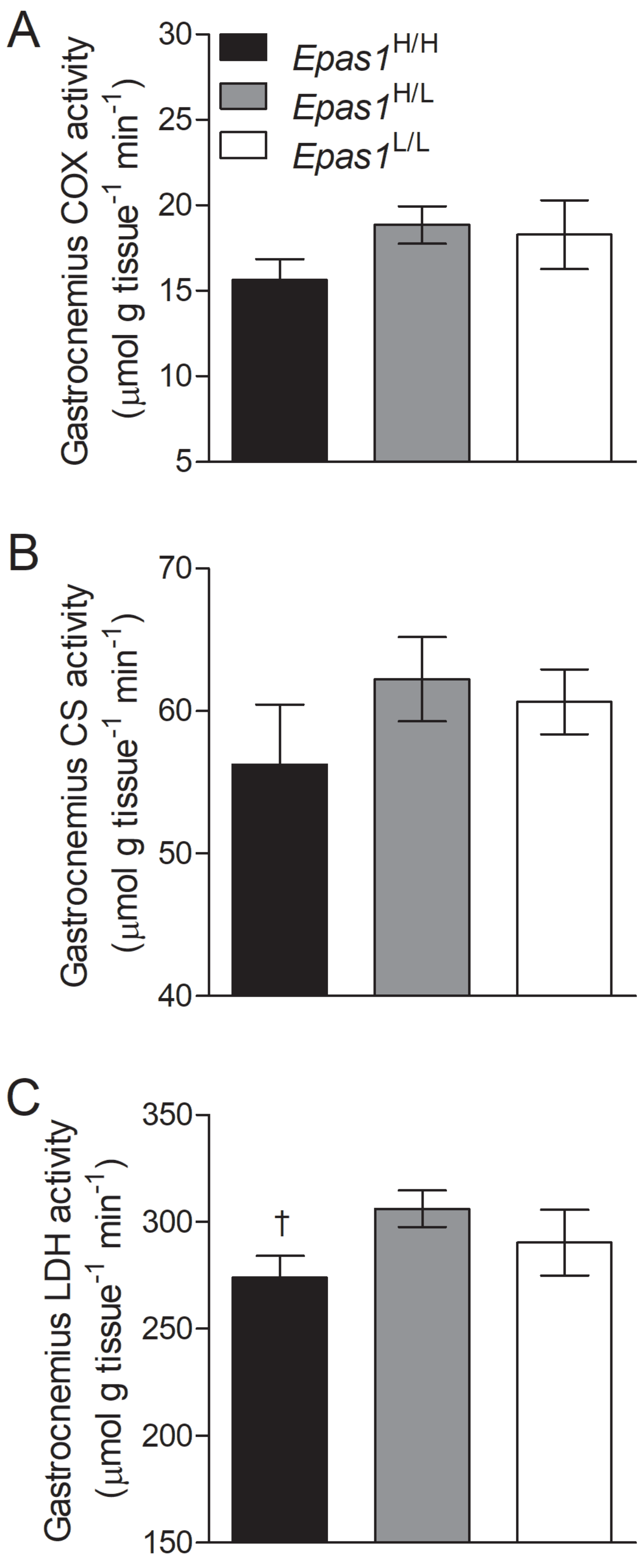

Figure S10. The activities of oxidative enzymes, i.e. A) cytochrome c oxidase, (COX) and B) citrate synthase (CS) in the gastrocnemius muscle were similar between deer mice with different *Epas1* genotypes, but C) lactate dehydrogenase (LDH) activity appeared to be lower in mice that were homozygous for the highland *Epas1* variant. † Significant difference in a post-hoc comparison between only *Epas1*$^{H/H}$ and *Epas1*$^{H/L}$ genotypes. n=16 *Epas1*$^{H/H}$, n=13 *Epas1*$^{H/L}$, and n=4 *Epas1*$^{L/L}$ variants.
