# Supplementary material for: Physiological and genomic evidence that selection on the transcription factor Epas1 has altered cardiovascular function in high-altitude deer mice
Source: PLoS Genet. 2019 Nov 7;15(11):e1008420. doi: 10.1371/journal.pgen.1008420 (PMC6837288; doi:10.1371/journal.pgen.1008420)
Supplement: S11 Fig — Deer mice with different Epas1 genotypes exhibited similar ventilatory responses to increasingly severe levels of acute hypoxia. Ventilation was quantified via A) total ventilation, B) arterial oxygen saturation, C) breathing frequency, and D) tidal volume. Sample sizes: n = 26 Epas1H/H, n = 13 Epas1H/L, and n = 4 Epas1L/L variants. (PDF) [file pgen.1008420.s025.pdf]

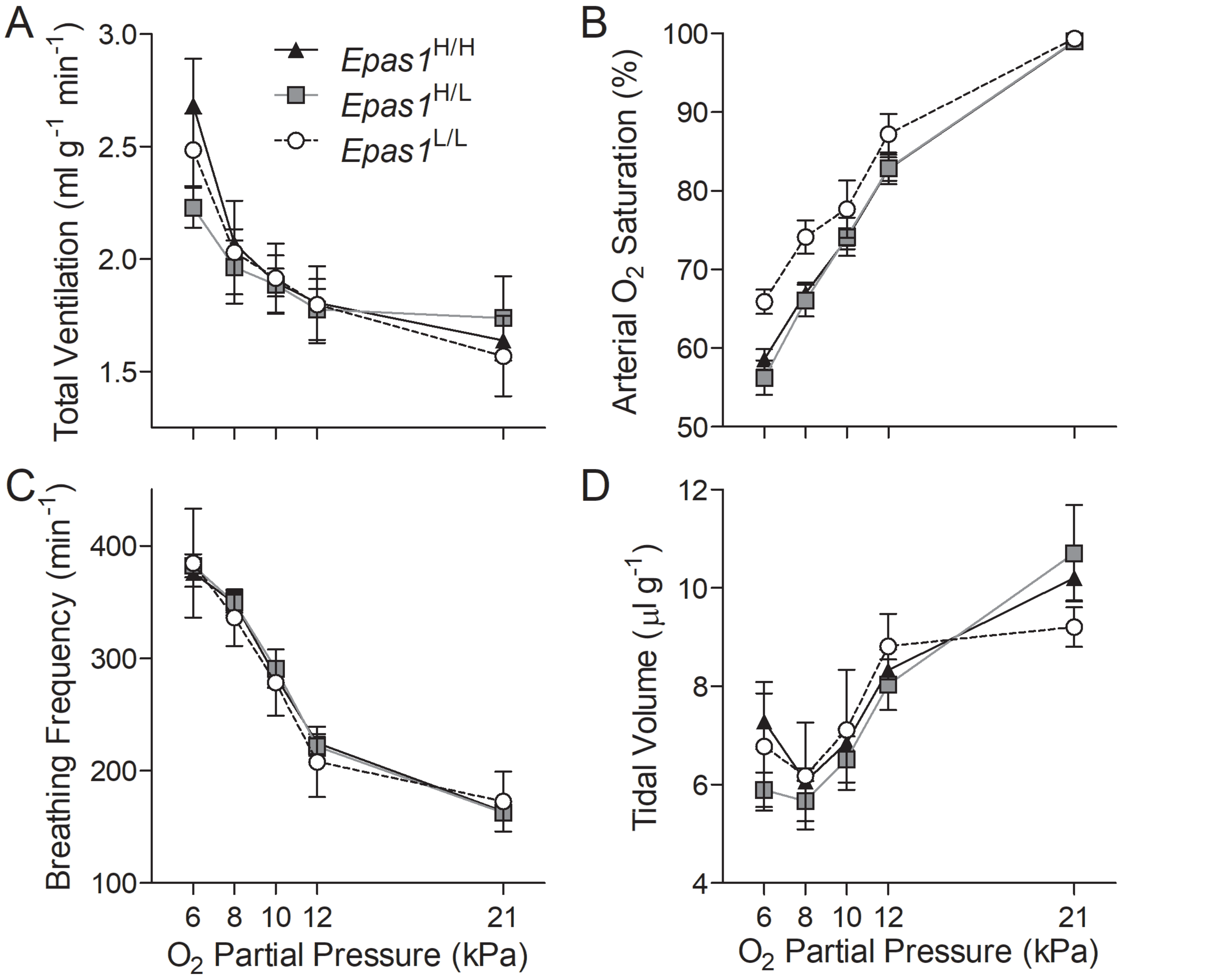


Figure S11. Deer mice with different *Epas1* genotypes exhibited similar ventilatory responses to increasingly severe levels of acute hypoxia. Ventilation was quantified via A) total ventilation, B) arterial oxygen saturation, C) breathing frequency, and D) tidal volume. Sample sizes: n=26 *Epas1*$^{H/H}$, n=13 *Epas1*$^{H/L}$, and n=4 *Epas1*$^{L/L}$ variants.
