# Supplementary material for: Physiological and genomic evidence that selection on the transcription factor Epas1 has altered cardiovascular function in high-altitude deer mice
Source: PLoS Genet. 2019 Nov 7;15(11):e1008420. doi: 10.1371/journal.pgen.1008420 (PMC6837288; doi:10.1371/journal.pgen.1008420)
Supplement: S12 Fig — Deer mice with different Epas1 genotypes exhibited similar declines in A) O2 consumption rate and B) body temperature in response to increasingly severe levels of acute hypoxia, and similar increases in C) ventilatory equivalent for O2 and D) pulmonary O2 extraction. Sample sizes: n = 26 Epas1H/H, n = 13 Epas1H/L, and n = 4 Epas1L/L variants. (PDF) [file pgen.1008420.s026.pdf]

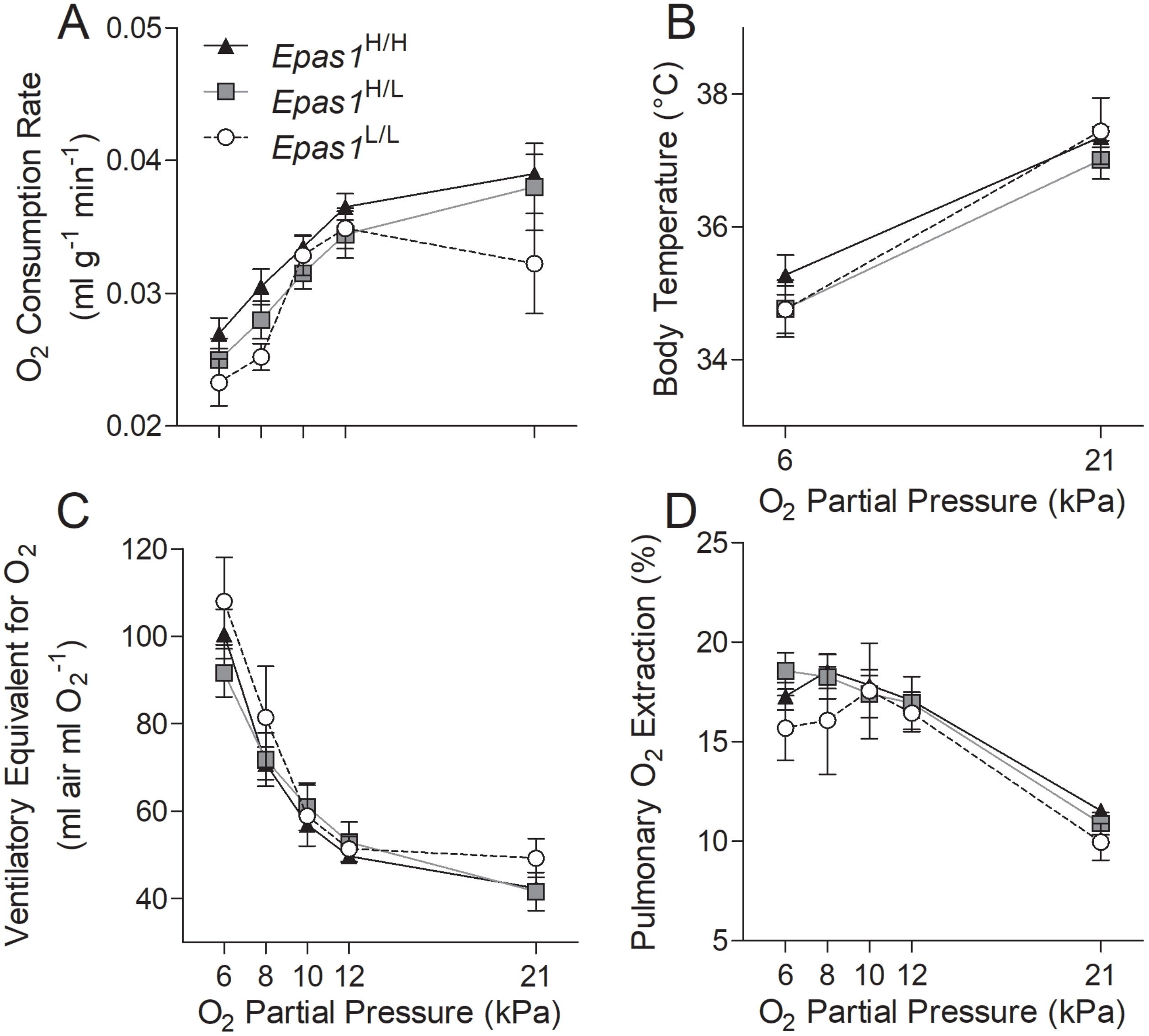

Figure S12. Deer mice with different *Epas1* genotypes exhibited similar declines in A) O_2 consumption rate and B) body temperature in response to increasingly severe levels of acute hypoxia, and similar increases in C) ventilatory equivalent for O_2 and D) pulmonary O_2 extraction. Sample sizes: n=26 *Epas1*$^{H/H}$, n=13 *Epas1*$^{H/L}$, and n=4 *Epas1*$^{L/L}$ variants.
