# Supplementary material for: Physiological and genomic evidence that selection on the transcription factor Epas1 has altered cardiovascular function in high-altitude deer mice
Source: PLoS Genet. 2019 Nov 7;15(11):e1008420. doi: 10.1371/journal.pgen.1008420 (PMC6837288; doi:10.1371/journal.pgen.1008420)
Supplement: S13 Fig — Deer mice that were homozygous for the highland Epas1 variant exhibited a significantly greater increase in heart rate from normoxia (21 kPa O2) to environmentally realistic levels of hypoxia at 4300 m elevation (12 kPa O2). Measurements were made using a MouseOx Plus collar. * A significant pairwise difference between Epas1H/H and Epas1H/L mice. n = 26 Epas1H/H, n = 13 Epas1H/L, and n = 4 Epas1L/L variants. (PDF) [file pgen.1008420.s027.pdf]

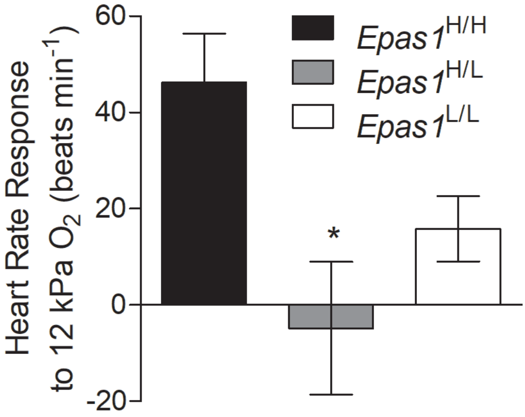

Figure S13. Deer mice that were homozygous for the highland *Epas1* variant exhibited a significantly greater increase in heart rate from normoxia (21 kPa O_2) to environmentally realistic levels of hypoxia at 4300 m elevation (12 kPa O_2). Measurements were made using a MouseOx Plus collar. * A significant pairwise difference between *Epas1*$^{H/H}$ and *Epas1*$^{H/L}$ mice. n=26 *Epas1*$^{H/H}$, n=13 *Epas1*$^{H/L}$, and n=4 *Epas1*$^{L/L}$ variants.
