# Supplementary material for: Physiological and genomic evidence that selection on the transcription factor Epas1 has altered cardiovascular function in high-altitude deer mice
Source: PLoS Genet. 2019 Nov 7;15(11):e1008420. doi: 10.1371/journal.pgen.1008420 (PMC6837288; doi:10.1371/journal.pgen.1008420)
Supplement: S14 Fig — The folded 2-dimensional site frequency spectra (2d-SFS) for deer mice from (A) Mt. Evans, CO, and Merced, CA, and (B) Mt. Evans, CO, and Lincoln, NE. For each pair of populations, we show the empirical 2d-SFS from whole exome data and the maximum likelihood 2d-SFS for demographic models with no migration, one migration rate, and two migration rates. Residuals reflect the overall fit of the model to the empirical data, where red indicates an overestimation of the number of SNPs by the model and blue reflects an underestimation. (PDF) [file pgen.1008420.s028.pdf]

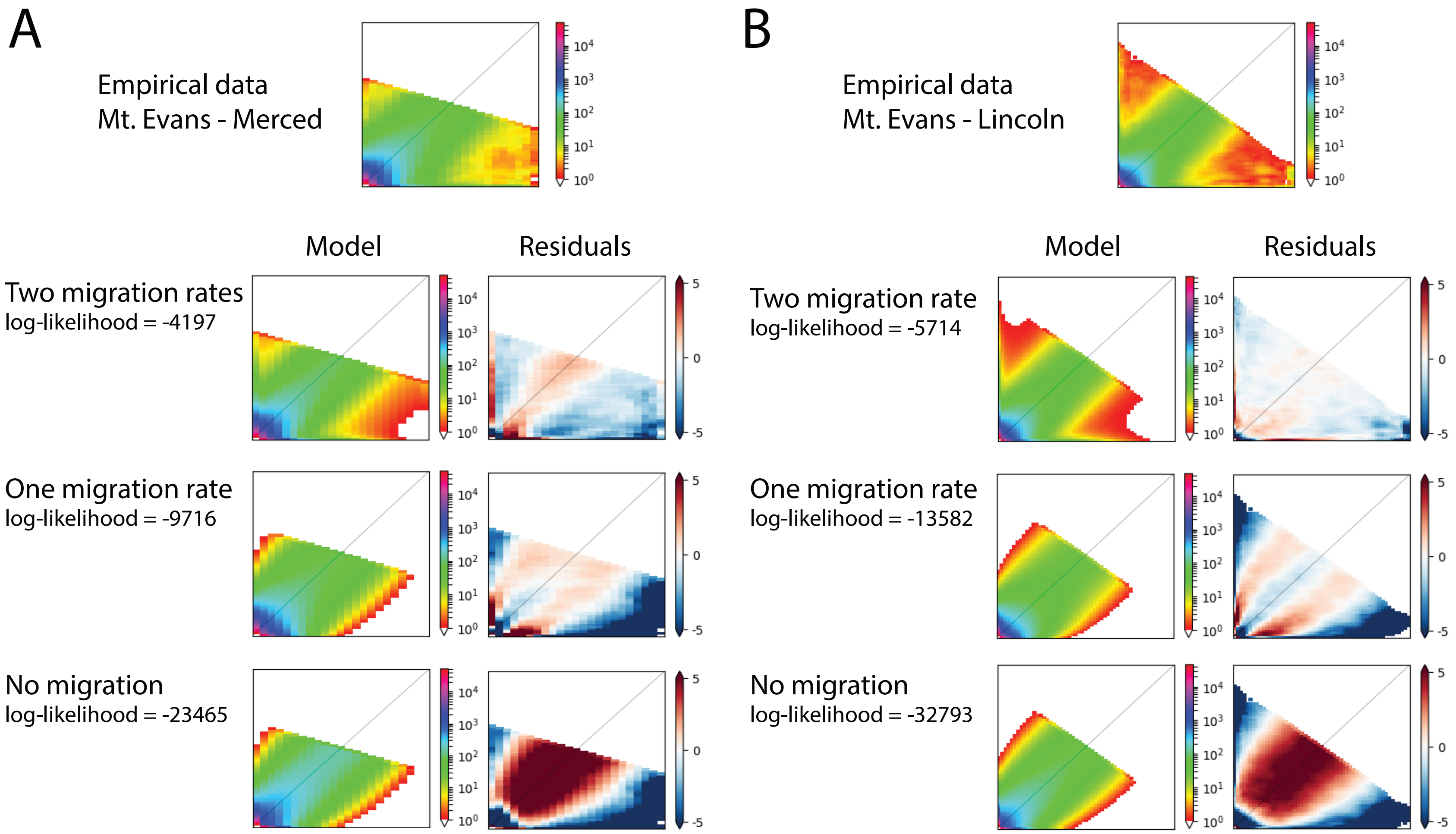

Figure S14. The folded 2-dimensional site frequency spectra (2d-SFS) for deer mice from (A) Mt. Evans, CO, and Merced, CA, and (B) Mt. Evans, CO, and Lincoln, NE. For each pair of populations, we show the empirical 2d-SFS from whole exome data and the maximum likelihood 2d-SFS for demographic models with no migration, one migration rate, and two migration rates. Residuals reflect the overall fit of the model to the empirical data, where red indicates an overestimation of the number of SNPs by the model and blue reflects an underestimation.
