# Supplementary material for: Physiological and genomic evidence that selection on the transcription factor Epas1 has altered cardiovascular function in high-altitude deer mice
Source: PLoS Genet. 2019 Nov 7;15(11):e1008420. doi: 10.1371/journal.pgen.1008420 (PMC6837288; doi:10.1371/journal.pgen.1008420)
Supplement: S15 Fig — A) Density plot of population branch statistic (PBS) values calculated for Mount Evans, using Lincoln and Merced populations as outgroups, from 10,000 SNPs simulated under modeled demography. Values for the mean (green vertical dashed line), 99th (blue vertical dotted line), and 99.9th (red dash-dotted line) percentiles of the simulated distribution are shown. B) Density plot of empirical PBS values (same as in S4 Fig), but with significance thresholds based on simulated 99th and 99.9th percentile shown. (PDF) [file pgen.1008420.s029.pdf]

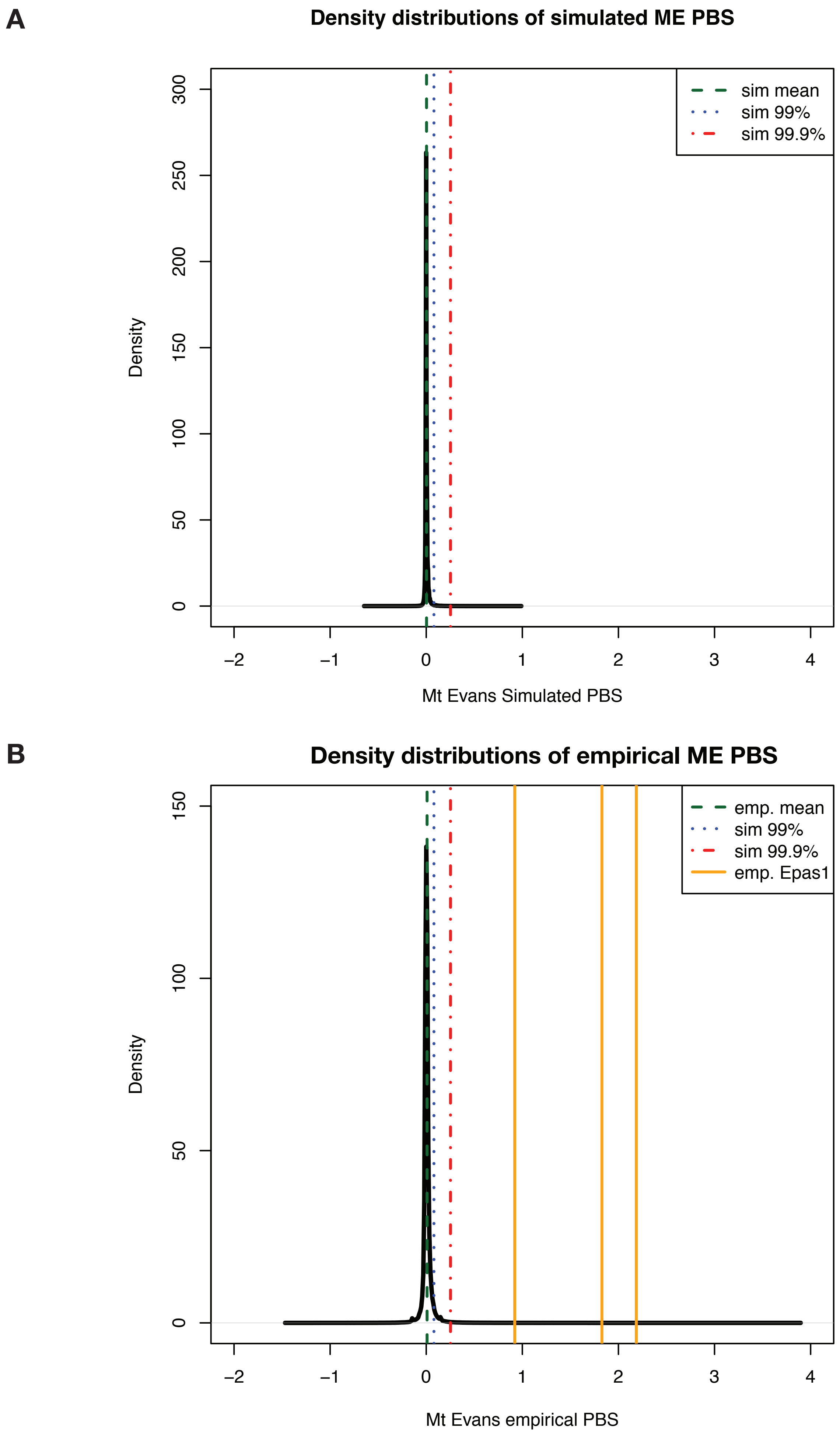

Figure S15. A) Density plot of population branch statistic (PBS) values calculated for Mount Evans, using Lincoln and Merced populations as outgroups, from 10,000 SNPs simulated under modeled demography. Values for the mean (green vertical dashed line), 99th (blue vertical dotted line), and 99.9th (red dash-dotted line) percentiles of the simulated distribution are shown. B) Density plot of empirical PBS values (same as in Figure S4), but with significance thresholds based on simulated 99th and 99.9th percentile shown.
