# Supplementary material for: Physiological and genomic evidence that selection on the transcription factor Epas1 has altered cardiovascular function in high-altitude deer mice
Source: PLoS Genet. 2019 Nov 7;15(11):e1008420. doi: 10.1371/journal.pgen.1008420 (PMC6837288; doi:10.1371/journal.pgen.1008420)
Supplement: S2 Fig — Principal components analysis of Mount Evans (n = 48), Lincoln (n = 37), and California (n = 15) mice, based on genotypes from 296,196 exome-wide LD-pruned SNPs with no missing data. (PDF) [file pgen.1008420.s016.pdf]

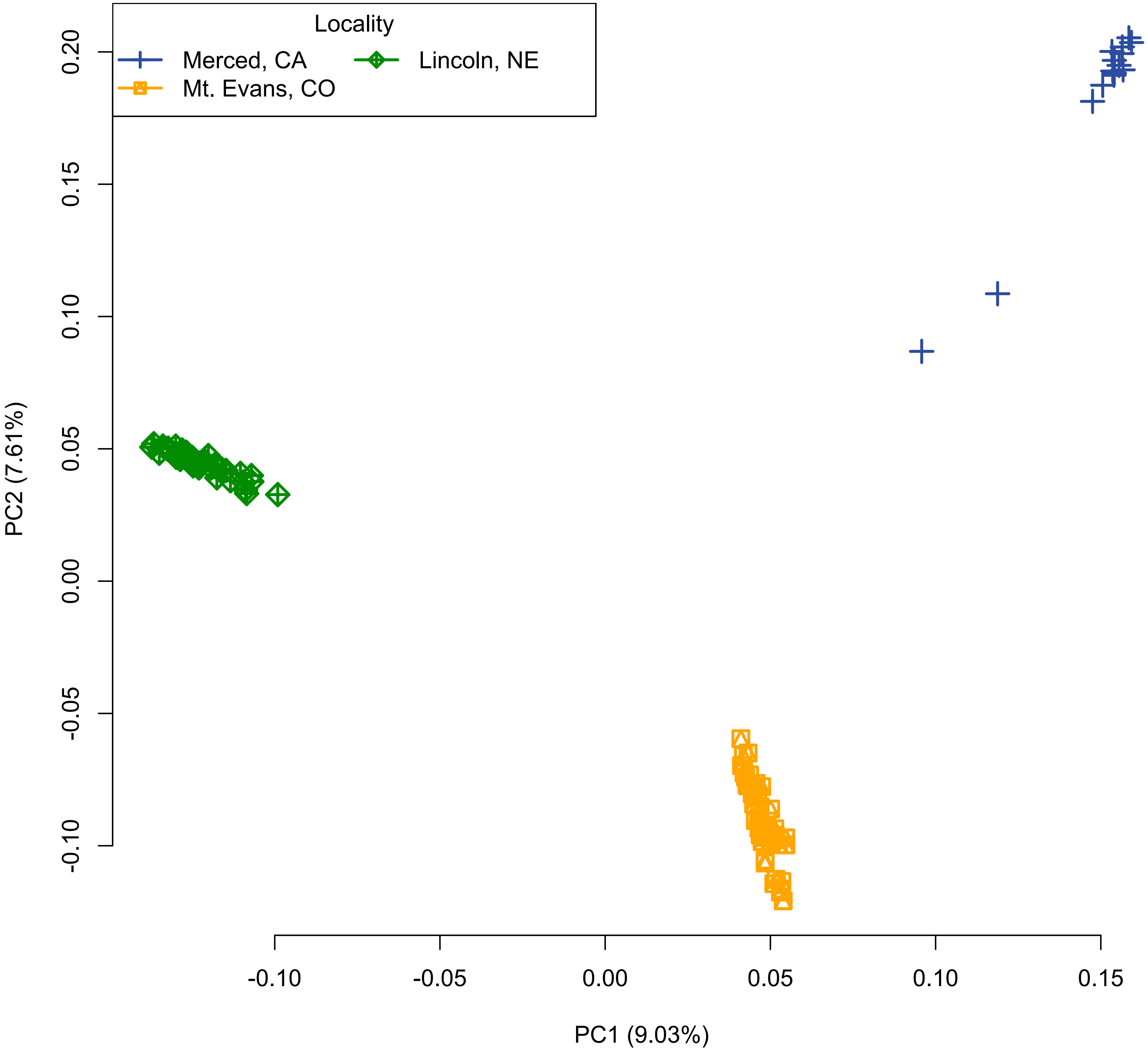

Figure S2. Principal components analysis of Mount Evans (n=48), Lincoln (n=37), and California (n=15) mice, based on genotypes from 296,196 exome-wide LD-pruned SNPs with no missing data.
